# Gemcitabine and Irinotecan as First-Line Therapy for Carcinoma of Unknown Primary: Results of a Multicenter Phase II Trial

**DOI:** 10.1371/journal.pone.0039285

**Published:** 2012-07-17

**Authors:** Shernan G. Holtan, Preston D. Steen, Nathan R. Foster, Charles Erlichman, Fabiola Medeiros, Matthew M. Ames, Stephanie L. Safgren, David L. Graham, Robert J. Behrens, Matthew P. Goetz

**Affiliations:** 1 Mayo Clinic Rochester, Rochester, Minnesota, United States of America; 2 Sanford Roger Maris Cancer Center, Fargo, North Dakota, United States of America; 3 Carle Cancer Center CCOP, Urbana, Illinois, United States of America; 4 Iowa Oncology Research Association CCOP, Des Moines, Iowa, United States of America; University Clinic of Navarra, Spain

## Abstract

Metastatic carcinoma of unknown primary (CUP) has a very poor prognosis, and no standard first-line therapy currently exists. Here, we report the results of a phase II study utilizing a combination of gemcitabine and irinotecan as first-line therapy. Treatment was with gemcitabine 1000 mg/m^2^ and irinotecan 75 mg/m^2^ weekly times four on a six week cycle (Cohort I). Due to excessive toxicity, the dose and schedule were modified as follows: gemcitabine 750 mg/m^2^ and irinotecan 75 mg/m^2^ given weekly times three on a four week cycle (Cohort II). The primary endpoint was the confirmed response rate (CR + PR). Secondary endpoints consisted of adverse events based upon the presence or absence of the UDP glucuronosyltransferase 1 family, polypeptide A1*28 (UGT1A1*28) polymorphism, time to progression, and overall survival. Thirty-one patients were enrolled with a median age of 63 (range: 38–94), and 26 patients were evaluable for efficacy. Significant toxicity was observed in Cohort 1, characterized by 50% (7/14) patients experiencing a grade 4+ adverse event, but not in cohort II. The confirmed response rate including patients from both cohorts was 12% (95% CI: 2–30%), which did not meet the criteria for continued enrollment. Overall median survival was 7.2 months (95% CI: 4.0 to 11.6) for the entire cohort but notably longer in cohort II than in cohort I (9.3 months (95% CI: 4.1 to 12.1) versus 4.0 months (95% CI: 2.2 to 15.6)). Gemcitabine and irinotecan is not an active combination when used as first line therapy in patients with metastatic carcinoma of unknown primary. Efforts into developing novel diagnostic and therapeutic approaches remain important for improving the outlook for this heterogeneous group of patients.

**Trial Registration:**

ClinicalTrials.gov NCT00066781

## Introduction

Patients with carcinoma of unknown primary (CUP) have metastatic cancer for which a primary site cannot be determined despite an algorithmic diagnostic approach including physical examination, imaging, endoscopy, and tumor biopsy with immunohistochemistry (IHC) [1]. Minimum criteria have been proposed for evaluation and treatment of CUP, including emphasis on identifying subsets of patients who have chemosensitive malignancies and a chance at cure, such as females with peritoneal carcinomatosis [2]. If a primary site can be determined, those patients may be able to receive tailored tumor-specific chemotherapy and have improved survival [3]. However, extensive evaluation can be time consuming, costly, and yield informative results in only a minority of patients [4]. Whole body 2′-[(18)F]fluoro-2′-deoxyglucose (FDG) PET/CT scanning improves the detection rates of primary tumors but has not been shown to improve survival [5]. Dealing with the uncertainty related to this diagnosis despite exhaustive testing is both difficult for patients and challenging for oncologists [6]. Improved diagnostic and treatment strategies are needed.

Several early phase II studies of broadly-acting combination chemotherapeutic regimens have yielded response rates in the 30–50% range (summarized by Greco and Pavlidis, 2009) [7], although these studies sometimes included patients with a favorable prognosis, including those with a single metastatic site, women with axillary adenopathy only, women with peritoneal carcinomatosis, squamous cell carcinoma isolated to cervical or inguinal lymph nodes, and men with blastic bony lesions [8], [9]. For example, when those favorable prognosis patients were excluded from analysis in a phase II trial of paclitaxel and carboplatin in CUP, the response rate decreased from 38.7% to 15.1%. True CUP, where the patient does not fit into one of the favorable categories, portends a very poor prognosis, with a median survival of approximately 6–10 months [10], [11]. This group is inherently heterogeneous, and as a result, defining optimal first line therapy has been difficult.

In the salvage setting, the combination of gemcitabine and irinotecan showed promise with 53% of patients deriving clinical benefit (10% overall response with 43% disease stability) from this combination [12]. Given these encouraging results and the relatively small chance of tumor response (around 15%) in patients treated with platinum and taxane regimens in the first line setting, we sought to evaluate gemcitabine and irinotecan in previously untreated patients with CUP. Additionally, given the association with increased gastrointestinal and bone marrow toxicity in patients who carry the UDP glucuronosyltransferase 1 family, polypeptide A1*28 (UGT1A1*28) polymorphism treated with irinotecan, we completed a parallel translational pharmacogenomics study [13]. Herein, we report the results of our multicenter phase II trial.

**Figure 1:**
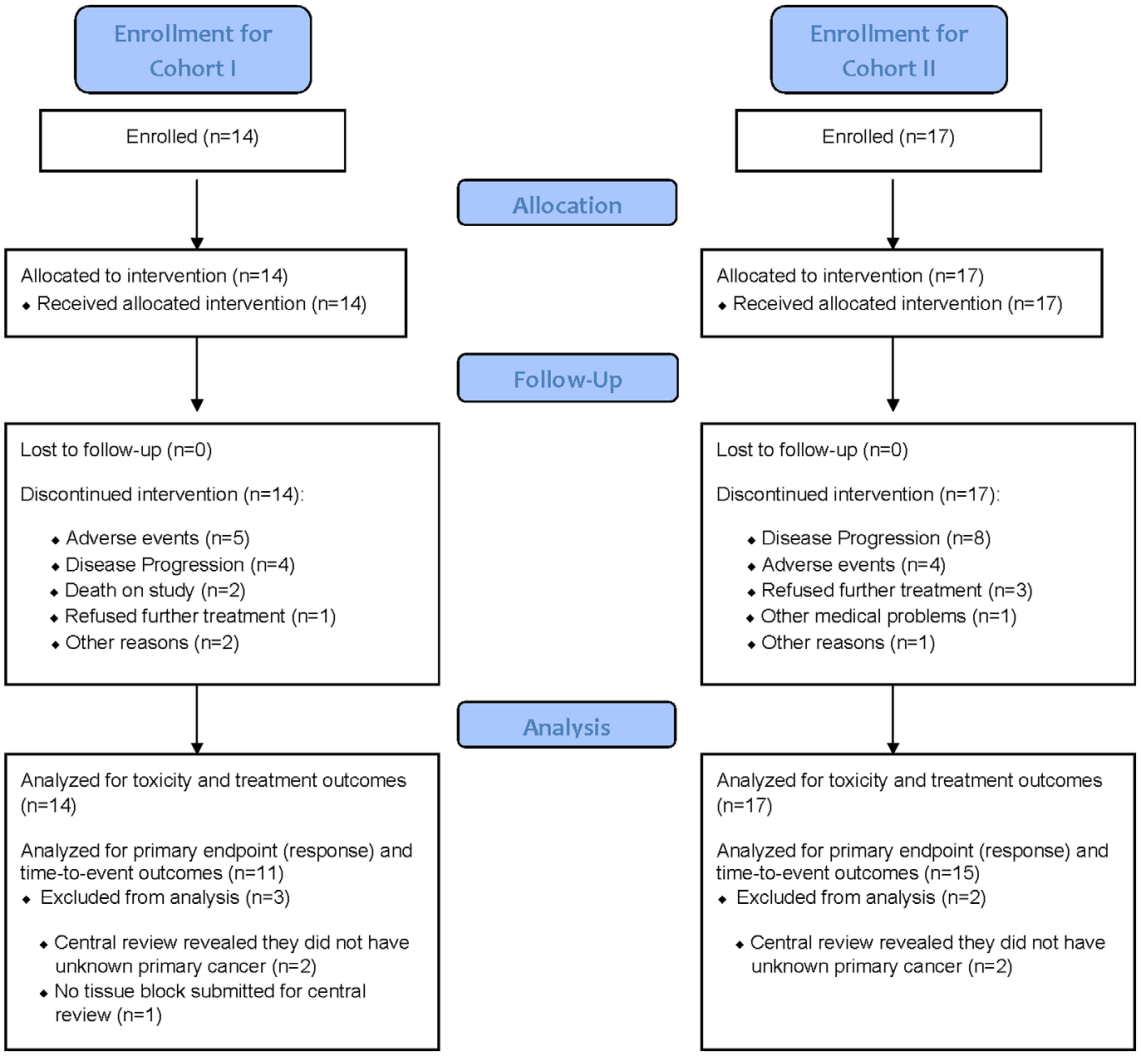
CONSORT diagram.

**Table 1:** Patient Characteristics.

	Cohort I (N = 14)	Cohort II (N = 17)	Total (N = 31)
**Age**			
Mean (SD)	62 (9)	61 (15)	62 (12)
Median	63	63	63
Range	(44–77)	(38–94)	(38–94)
**Performance Score**			
0	6 (43%)	8 (47%)	14 (45%)
1	8 (57%)	9 (53%)	17 (55%)
**Gender**			
Female	6 (43%)	8 (47%)	14 (45%)
Male	8 (57%)	9 (53%)	17 (55%)
**Prior Surgery**			
Yes	9 (64%)	7 (41%)	16 (52%)
No	5 (36%)	10 (59%)	15 (48%)
**Race**			
White	13 (93%)	15 (88%)	28 (90%)
Black or African American	0 (0%)	1 (6%)	1 (3%)
Asian	0 (0%)	1 (6%)	1 (3%)
Not reported: patient refused or not available	1 (7%)	0 (0%)	1 (3%)
**Tumor Grade Group**			
Missing	1 (7%)	2 (12%)	3 (10%)
Low (grade 1 or 2)	3 (21%)	4 (24%)	7 (23%)
High (grade 3 or 4)	10 (71%)	11 (65%)	21 (68%)
**Histology**			
Adenocarcinoma	8 (57%)	8 (47%)	16 (52%)
Poorly Differentiated Adenocarcinoma	2 (14%)	6 (35%)	8 (26%)
Poorly Differentiated Carcinoma	3 (21%)	2 (12%)	5 (16%)
Poorly Differentiated Squamous Carcinoma	1 (7%)	1 (6%)	2 (7%)
UGT1A1 *28 ** Genotype**			
7/7 TA repeats	3 (21%)	3 (18%)	6 (19%)
6/7 TA repeats	3 (21%)	8 (47%)	11 (36%)
6/6 TA repeats	8 (57%)	6 (35%)	14 (45%)

## Methods

The protocol for this trial and supporting CONSORT checklist are available as supporting information; see Checklist S1 and Protocol S1.

### Participants

This phase II clinical trial was initiated through the North Central Cancer Treatment Group in 2004. Patients were eligible for enrollment if they had histologically confirmed CUP after central pathology review, chest imaging, abdomen/pelvis CT scan, directed evaluation of the symptomatic areas, mammogram in women, colonoscopy in patients with liver metastases, and measurable disease by RECIST criteria [14]. Patients who fell into favorable prognostic categories (including those with a single metastatic site, women with axillary adenopathy only, women with peritoneal carcinomatosis, squamous cell carcinoma isolated to cervical or inguinal lymph nodes, men with blastic bony lesions, and neuroendocrine carcinomas) were excluded. Baseline performance status of ECOG 0, 1 or 2, adequate bone marrow function (hemoglobin >9 g/dL, neutrophil count >1.5/uL, platelet count >100,000/uL), and normal kidney and liver function tests were required. Patients could not have received prior chemotherapy. Additionally, pregnant or lactating women, those with severe immune compromise such as with HIV infection, severe comorbid illnesses, active other malignancy (except non-melanotic skin cancer or carcinoma *in situ* of the cervix), and those with untreated brain metastases were excluded from enrollment.

### Ethics

This study was approved by the Mayo Clinic Institutional Review Board as well as the institutional review board of all participating North Central Cancer Treatment Group sites (Sanford Roger Maris Cancer Center, Carle Cancer Center, Iowa Oncology Research Association, Siouxland Hematology-Oncology Associates, Metro-MN Community Clinical Oncology Program (CCOP), Dayton CCOP, Essentia Duluth CCOP, Cedar Rapids Oncology Project CCOP, Wichita CCOP, Missouri Valley Cancer Consortium, Omaha, Cedar Rapids Oncology Project CCOP, addresses of all listed in Acknowledgments). All patients were required to give written informed consent before participation.

**Figure 2:**
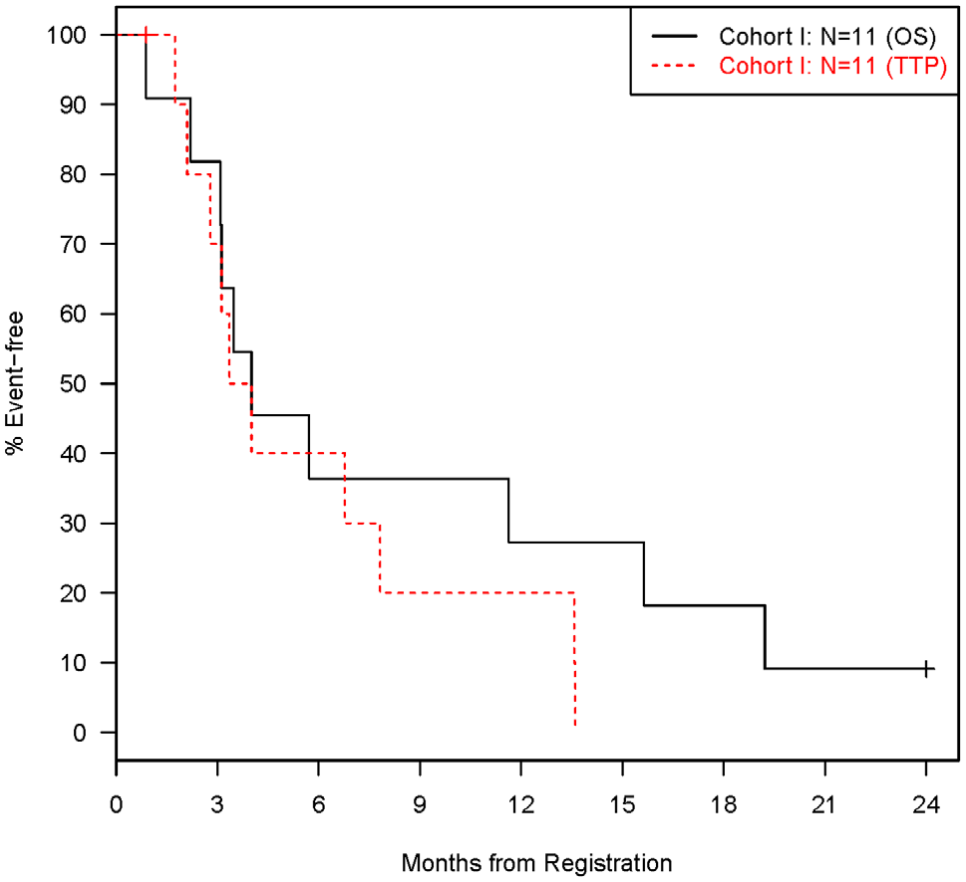
Distributions of Time to Disease Progression and Death (Cohort I: N = 11).

**Figure 3:**
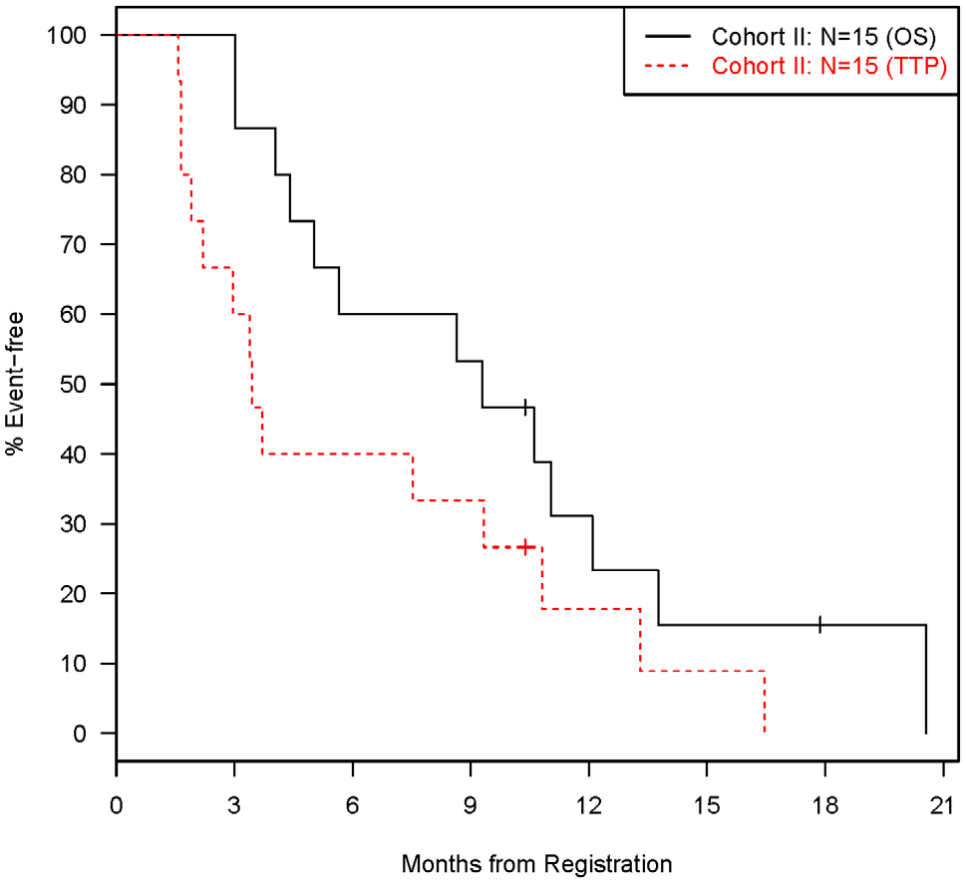
Distributions of Time to Disease Progression and Death (Cohort II: N = 15).

### Treatment

Cohort I patients received 1000 mg/m^2^ of gemcitabine on days 1, 8, 15, 22 and 75 mg/m^2^ of irinotecan on days 1, 8, 15, 22. The cycle length was 6 weeks which included a 2 week rest period. Due to slow accrual and high toxicity rates in Cohort I, the study was redesigned, the dose was reduced, and cycle length was modified. Cohort II patients received a schedule that consisted of 750 mg/m^2^ of gemcitabine on days 1, 8, 15 and 75 mg/m2 of irinotecan on days 1, 8, 15. The cycle length was 4 weeks which included a 1 week rest period. All patients from both cohorts were to continue treatment until cancer progression, unacceptable toxicity, or patient refusal.

### Follow-up Evaluations

While receiving protocol treatment, visits with an oncologist were required every cycle for both cohorts (cohort I: 6 weeks, cohort II: 4 weeks), but were otherwise monitored with weekly blood testing as deemed clinically appropriate by their healthcare providers. For both cohorts, tumor measurements were to be obtained immediately before the anticipated third cycle, and every other cycle thereafter unless more frequent assessments were required to confirm a tumor response. RECIST criteria were used to define a confirmed tumor response [14]. Adverse events were recorded with the National Cancer Institute’s Common Toxicity Criteria, version 3.0, and tracked throughout the study period. Patients no longer receiving treatment were followed every 3 months for a maximum of 2 years from study entry.

**Table 2:** Cohort I: Percent of Targeted Dose (relative to targeted dose at start of treatment) for patients receiving Gemcitabine and Irinotecan.

Cycle	No. of Patients Treated	Gemcitabine		Irinotecan	
		Mean (SD)	Median (Range)	Mean (SD)	Median (Range)
1	14	80 (23)	75 (25–102)	77 (26)	75 (13–102)
2	10	77 (24)	83 (38–102)	66 (30)	55 (24–100)
3	6	73 (26)	75 (38–100)	61 (31)	49 (23–100)
4	4	78 (29)	88 (38–100)	67 (38)	73 (24–100)
5	3	91 (14)	98 (75–100)	81 (30)	96 (47–100)
6	2	56 (27)	56 (38–75)	49 (37)	49 (23–75)

**Table 3:** Cohort I: Frequency of Dose Reductions for patients receiving Gemcitabine and Irinotecan.

Cycle	No. of Patients Treated	Gemcitabine, N (%)		Irinotecan, N (%)	
		Full dose^1^	Reduced dose	Full dose^1^	Reduced dose
1	14	6 (43%)	8 (57%)	6 (43%)	8 (57%)
2	10	4 (40%)	6 (60%)	4 (40%)	6 (60%)
3	6	2 (33%)	4 (67%)	2 (33%)	4 (67%)
4	4	2 (50%)	2 (50%)	2 (50%)	2 (50%)
5	3	1 (33%)	2 (67%)	1 (33%)	2 (67%)
6	2	0 (0%)	2 (100%)	0 (0%)	2 (100%)

1 Defined as 98% or more of the targeted dose.

### Primary Endpoint

The primary endpoint for both cohorts was the confirmed response rate. All patients who were eligible, signed a consent form, and started treatment were evaluable for this endpoint. The confirmed response rate was calculated as the number of patients who had a confirmed response (i.e. 2 consecutive responses (CR or PR) at least 4 weeks apart) divided by the total number of evaluable patients.

### Cohort I Statistical Design for Primary Endpoint

A three-outcome phase II study design [15] with an interim analysis was used to test whether there was sufficient evidence to determine if the confirmed response rate was at least 40% (ie, clinically promising) versus at most 20% (i.e., clinically inactive). This study was designed to have 85% power to detect a confirmed response rate of 40%, with a.05 level of significance. At least 4 confirmed responses in the first 17 evaluable patients (interim analysis), would expand patient enrollment to 37 evaluable patients, unless undue toxicity was observed. If the study did reach the full accrual of 37 evaluable patients, 3 conclusions were possible: Not promising (10 or fewer confirmed responses); Inconclusive (11 confirmed responders); and Promising (12 or more confirmed responses). A confidence interval for the confirmed tumor response rate was calculated using the standard binomial method.

**Table 4:** Cohort II: Percent of Targeted Dose (relative to targeted dose at start of treatment)for patients receiving Gemcitabine and Irinotecan.

Cycle	No. of Patients Treated	Gemcitabine		Irinotecan	
		Mean (SD)	Median (Range)	Mean (SD)	Median (Range)
1	17	90 (16)	100 (67–101)	86 (21)	100 (33–100)
2	12	97 (10)	100 (66–102)	95 (12)	100 (66–102)
3	8	99 (3)	100 (92–101)	99 (3)	99 (93–101)
4	8	89 (18)	98 (53–100)	89 (18)	97 (53–100)
5	5	90 (12)	97 (75–100)	90 (12)	97 (74–100)
6	5	88 (16)	97 (64–100)	87 (18)	97 (60–100)

**Table 5:** Cohort II: Frequency of Dose Reductions for patients receiving Gemcitabine and Irinotecan.

Cycle	No. of Patients Treated	Gemcitabine, N (%)		Irinotecan, N (%)	
		Full dose^1^	Reduced dose	Full dose^1^	Reduced dose
1	17	12 (71%)	5 (29%)	11 (65%)	6 (35%)
2	12	10 (83%)	2 (17%)	10 (83%)	2 (17%)
3	8	7 (88%)	1 (12%)	6 (75%)	2 (25%)
4	8	4 (50%)	4 (50%)	3 (37.5%)	5 (62.5%)
5	5	2 (40%)	3 (60%)	2 (40%)	3 (60%)
6	5	2 (40%)	3 (60%)	2 (40%)	3 (60%)

1 Defined as 98% or more of the targeted dose.

### Cohort II Statistical Design for Primary Endpoint

A three-outcome phase II study design [15] with an interim analysis was used to test whether there was sufficient evidence to determine if the confirmed response rate was at least 40% (ie, clinically promising) versus at most 20% (i.e., clinically inactive). This study was designed to have 87% power to detect a confirmed response rate of 40%, with a.10 level of significance. At least 3 confirmed responses in the first 13 evaluable patients (interim analysis), would expand patient enrollment to 25 patients. Otherwise, 2 or fewer confirmed responses in the first 13 evaluable patients would permanently close the trial due to a lack of efficacy. If the study did reach the full accrual of 25 evaluable patients, 3 conclusions were possible: Not promising (6 or fewer confirmed responses); Inconclusive (7 confirmed responders); and Promising (8 or more confirmed responses). A confidence interval for the confirmed tumor response rate was calculated using the standard binomial method.

**Table 6:** Cohort I: The maximum grade adverse events for all cycles of therapy, regardless of attribution.

Adverse Event	Grade 3 N (%)	Grade 4 N (%)
**Gastrointestinal**		
Nausea	2 (14%)	0 (0%)
Colitis	0 (0%)	2 (14%)
**Hematology**		
Thrombocytopenia	2 (14%)	0 (0%)
Leukopenia	6 (43%)	1 (7%)
Neutropenia	4 (29%)	1 (7%)
**Pulmonary**		
Dyspnea	1 (7%)	1 (7%)
**Cardiovascular**		
Ischemia/Infarction	0 (0%)	1 (7%)
**Hepatic**		
Bilirubin	0 (0%)	1 (7%)
**Other**		
Fatigue	3 (21%)	0 (0%)

N = 14 patients evaluable for adverse events.

### Secondary Endpoints

Secondary endpoints for both cohorts included adverse events, time to disease progression, and overall survival. Adverse events were summarized in a tabular manner as the maximum grade for a given type of event for each patient. All grade 4 or 5 adverse events (regardless of attribution) are reported, along with grade 3 adverse events that occurred in at least 10% of patients (i.e. at least 2 patients) for each cohort. Time to disease progression was defined as the time from study entry to disease progression, where patients with no progression were censored on their last tumor assessment date. Overall survival was defined as the time from study entry to death from any cause, where patients that were still alive were censored on their last follow-up date. Time-to-event data was censored at 2 years since the study protocol had a maximum follow-up of 2 years. Kaplan-Meier methodology [16] was used to describe the distributions of time to disease progression and overall survival.

**Table 7:** Cohort II: The maximum grade adverse events for all cycles of therapy, regardless of attribution.

Adverse Event	Grade 3 N (%)	Grade 4 N (%)
**Hematology**		
Thrombocytopenia	2 (12%)	0 (0%)
Neutropenia	4 (24%)	0 (0%)
**Gastrointestinal**		
Nausea	2 (12%)	0 (0%)
Diarrhea	3 (18%)	0 (0%)
**Hepatic**		
Bilirubin	2 (12%)	0 (0%)
**Other**		
Fatigue	2 (12%)	0 (0%)
Febrile Neutropenia	2 (12%)	0 (0%)
Hyponatremia	2 (12%)	0 (0%)
Renal Failure	0 (0%)	1 (6%)

N = 17 patients evaluable for adverse events.

## Results

### Patient Characteristics

#### Cohort I

Between February 20, 2004 and August 5, 2005, 14 patients were enrolled to Cohort I (Figure 1). The patient characteristics are outlined in Table 1. Eight (57%) of the patients were men, median age was 63 (range 44–77), 10 (71%) had a high grade tumor by histologic examination, 8 (57%) patients were 6/6 TA repeats (i.e., negative for the UGT1A1*28 polymorphism, where a seventh TA sequence is present in the TATA sequence of the *UGT1A1* promoter). Patients had predominant disease (patients may have more than one category) for the following sites: Liver (6), Lung (4), Soft Tissue (1), Bone (2), and Other (8).

#### Cohort II

Between December 16, 2005 and August 31, 2007, 17 patients were enrolled to Cohort II (Figure 1). The patient characteristics are outlined in Table 1. Nine (53%) of the patients were men, median age was 63 (range 38–94), 11 (65%) had a high grade tumor by histologic examination, 6 (35%) patients were 6/6 TA repeats. Patients had predominant disease (patients may have more than one category) for the following sites: Liver (11), Lung (5), Soft Tissue (3), Bone (1), and Other (5).

### Outcomes and Estimation

#### Cohort I

Eleven patients were evaluable for response, which excludes 3 patients that were found ineligible because either the central pathology review revealed they did not have unknown primary cancer (n = 2) or no tissue block was submitted for central pathology review (n = 1; Figure 1). One patient was still alive at two years but had progressed. One patient died without disease progression. The other 9 patients have progressed and died. The overall confirmed response rate (1 PR) was 9% [95% confidence interval (CI): 0% to 41%], which did not meet our predefined criteria for success. The median survival (Figure 2) was 4.0 months (95% CI: 2.2 to 15.6 months) and the median time to progression (Figure 2) was 3.7 months (95% CI: 1.7 to 7.8 months).

#### Cohort II

Fifteen patients were evaluable for response, which excludes 2 patients that were found ineligible because the central pathology review revealed they did not have unknown primary cancer (Figure 1). Two patients are still alive (one has progressed and the other has not). The other 13 patients have progressed and died. The overall confirmed response rate (2 PR) was 13% [95% confidence interval (CI): 2% to 40%], which did not meet our predefined criteria for success. The median survival (Figure 3) was 9.3 months (95% CI: 4.1 to 12.1 months) and the median time to progression (Figure 3) was 3.4 months (95% CI: 1.6 to 9.3 months).

### Dose Intensity

#### Cohort I

A median of 2 cycles of therapy was given (range: 1–14). The percent of targeted dose administered for the first 6 cycles is shown in Table 2. The gemcitabine median stayed consistent from cycles 1 to 3, while the irinotecan median decreased from cycle 1 to 3. By cycle 4, only 4 patients were still receiving treatment. Of the 10 patients that were treated though cycle 2, 40% received the full dose for both gemcitabine and irinotecan (relative to the original targeted dose before treatment started). The dose reductions by cycle (first 6 cycles) are shown in Table 3.

#### Cohort II

A median of 2 cycles of therapy was given (range: 1–12). The percent of targeted dose administered for the first 6 cycles is shown in Table 4. Both the gemcitabine and irinotecan median dose stayed consistent from cycles 1 to 6. By cycle 6, 5 patients were still receiving treatment. Of the 12 patients that were treated through cycle 2, 83% received the full dose for both gemcitabine and irinotecan (relative to the original targeted dose before treatment started). The dose reductions by cycle (first 6 cycles) are shown in Table 5.

### Adverse Events

#### Cohort I

Fourteen patients were evaluable for adverse events. Thirteen (93%) patients experienced at least one grade 3+ adverse event. Seven (50%) patients experienced at least one grade 4+ adverse event, with two grade 5 adverse events (multi-organ failure, sudden death). Both grade 5 events were thought to be not related to study treatment. The most common hematological grade 3+ adverse event was leukopenia, experienced by 7 (50%) patients. There were no hematological treatment related deaths. The most commonly occurring grade 3+ non-hematological adverse events included fatigue (21%), nausea (14%), dyspnea (14%), and colitis (14%). Hematological and non-hematological adverse events are outlined in Table 6. Due to the high rates of grade 3/4 adverse events experienced in this cohort, the dose was reduced and the trial was re-designed for cohort II patients.

#### Cohort II

Seventeen patients were evaluable for adverse events. Eleven (65%) patients experienced at least one grade 3+ adverse event. One (6%) patient experienced at least one grade 4+ adverse event. No grade 5 events were reported. The most common hematological grade 3+ adverse events were thrombocytopenia (12%) and neutropenia (24%). The most commonly occurring grade 3+ non-hematological adverse events included nausea (12%), fatigue (12%), diarrhea (18%), bilirubin (12%), febrile neutropenia (12%), and hyponatremia (12%). Hematological and non-hematological adverse events are outlined in Table 7.

### Ancillary Analyses

Due to few individuals being evaluable for adverse events, conclusions regarding any difference in toxicity based upon the UGT1A1*28 polymorphism cannot be drawn.

## Discussion

### Interpretation

This regimen of gemcitabine and irinotecan did not meet our pre-defined criteria for efficacy in poor prognosis patients with CUP and, at standard doses, was toxic. The median survival was 7.2 months for the entire group, indicating the need for novel approaches beyond broad spectrum combination chemotherapy for this group of patients. In cohort II, where the regimen was adjusted for tolerability, the observed median survival was notably longer as compared to cohort I (9.3 months (95% CI: 4.1 to 12.1) vs. 4.0 months (95% CI: 2.2 to 15.6)), suggesting that greater dose intensity does not always lead to improved survival in cancer therapy [17].

### Generalizability

The external validity of these findings is strengthened by the multi-center design and implementation of this trial. Since patients with CUP are inherently heterogenous, the outcome of the trial would likely be similar were it to have been conducted in a different setting. Indeed, a phase II community-based, multicenter CUP trial conducted contemporaneously with this study yielded similar results to our cohort II. This clinical trial randomized patients to paclitaxel/carboplatin/etoposide (93 patients) versus gemcitibine and irinotecan (105 patients), both followed by gefitinib maintenance. In this trial, irinotecan was given at 100 mg/m^2^ IV and gemcitabine 1000 mg/m^2^ IV, both on days 1 and 8 of a 21 days cycle, yielding a response rate of 18% and median overall survival of 8.5 months [18].

### Overall Evidence

This clinical trial was conducted prior to routine addition of targeted therapy to cytotoxic regimens, a relatively recent revolution in cancer therapeutics. The search for targeted therapies that could rationally be applied to patients with CUP is ongoing. For example, expression of epidermal growth factor receptor (EGFR) is common in CUP [19]. Despite this phenomenon, the first therapeutic trial to incorporate an agent targeting the EGFR tyrosine kinase domain, gefitinib, did not appear to prevent progression of disease after responses were achieved with multiagent chemotherapy [18]. Additionally, expression of vascular endothelial growth factor (VEGF) is a very frequent occurrence in CUP, making disruption of angiogenesis pathways attractive [20]. Bevacizumab (VEGF receptor antibody) and erlotinib (second generation EGFR tyrosine kinase inhibitor), given in a phase II clinical trial for treatment-naïve or chemotherapy refractory metastatic CUP, maintained disease stability for 61% of the 47 patients enrolled on the study for a median of 4 months (range, 2 to 15+ months). Additionally, 5 patients had partial responses, and the median survival for this group was 7.4 months [21]. Since the combination of targeted agents was well-tolerated and showed encouraging results, the regimen was added to a chemotherapeutic backbone of paclitaxel and carboplatin in a phase II study of paclitaxel, carboplatin, bevacizumab, plus erlotinib. In this study, median overall survival was 12.6 months, a favorable result compared to previous trials [22]. Other studies utilizing a combination of targeted therapies along with chemotherapy are ongoing, including an NCCTG study evaluating paclitaxel, carboplatin, and the mTOR inhibitor, everolimus, (Clinicaltrials.gov identifier NCT00936702, assessed 11/3/11).

Improved diagnostic techniques may help personalize therapy for heterogeneous CUP patients. In 2008, the FDA approved the Pathwork® Tissue of Origin (Pathwork Diagnostics, Redwood City, CA) test in which molecular profiling classifies poorly differentiated cancers into more common subtypes based on microarray-determined patterns of gene expression [23]. The overall accuracy of this gene expression profiling tool approaches 90% [24]. Another platform, CancerTYPE ID (bioTheranostics®, Inc., San Diego, CA) which uses a reverse transcriptase-polymerase chain reaction (RT-PCR) assay molecular profiling, has shown 75% accuracy in determining actual latent primary tumor sites [25]. Retrospective analyses suggest that tailoring therapy to colon cancer-specific regimens when a high-probability colon cancer origin is detected via the CancerTYPE ID platform is associated with improved survival [26]. It is not yet known whether this type of technology will help tailor therapy and improve survival for all cancer subtypes. Prospective studies are underway to address this question [27]. A non-randomized phase II study of molecular profiling-guided systemic therapy for CUP has completed accrual and has now closed (ClinicalTrials.gov identifier NCT00737243, assessed 3/20/12).

Many diagnostic and therapeutic challenges remain in CUP. This multi-center phase II trial of gemcitabine and irinotecan in poor-risk CUP patients did not meet pre-defined response criteria for study continuation and was terminated after 31 patients were enrolled. Efforts into developing tailored approaches for these patients with heterogeneous malignancies are ongoing.

## Supporting Information

Checklist S1 **CONSORT Checklist.**
(DOC)

Protocol S1 **Trial Protocol.**
(PDF)
