# Supplementary material for: Gemcitabine and Irinotecan as First-Line Therapy for Carcinoma of Unknown Primary: Results of a Multicenter Phase II Trial
Source: PLoS One. 2012 Jul 17;7(7):e39285. doi: 10.1371/journal.pone.0039285 (PMC3398897; doi:10.1371/journal.pone.0039285)
Supplement: Protocol S1 — Trial Protocol. (PDF) [file pone.0039285.s002.pdf]

North Central Cancer Treatment Group

A Phase II Study of Gemcitabine (GEMZAR) and Irinotecan (CPT-11) in Previously Untreated Patients with Measurable Disease with Unknown Primary Carcinoma

For any communications regarding this protocol,
please call the protocol resource person on the following page.

Study Chairs: Matthew P. Goetz, M.D. (Research Base)*
Mayo Foundation
200 First Street SW
Rochester, MN 55905
Phone: (507) 284-2511
Fax: (507) 284-5280
E-mail: goetz.matthew@mayo.edu

Preston Steen, M.D. (NCCTG)
MeritCare Hospital CCOP
Roger Maris Cancer Center
820 4th Street North
Fargo, ND 58122-9988
Phone: (701) 234-2397
Fax: (701) 234-6444
E-mail: prestonsteen@meritcare.com

Study Cochairs: Charles Erlichman, M.D. (Research Base)
Matthew Ames, PhD†
Richard Weinshilboum, M.D. †
Marie Christine Aubry, M.D. (Research Base Pathologist) †
Timothy F. Drevyanko, M.D. (NCCTG Pathologist) †

Statistician: Nathan R. Foster, M.S.
(507/284-8803)†

† Study contributor not responsible for patient care.

*Investigator having NCI responsibility for this protocol.

**Document History**	**(Effective Date)**
Activation	February 20, 2004
Addendum 1	July 2, 2004
Addendum 2	March 18, 2005
Addendum 3	October 28, 2005
Addendum 4	November 23, 2005
Addendum 5	November 3, 2006
Update 1	November 3, 2006
Addendum 6	July 27, 2007
Addendum 7	July 11, 2008
Addendum 8	March 6, 2009

**Study Participants**	**Date Activated**
Entire NCCTG	February 20, 2004

NCI Version Date: June 19, 2008

Protocol Resource

	Questions:	Contact Name:
Add 2	Patient eligibility*, test schedule, treatment delays/interruptions/adjustments, dose modifications, adverse events	Paula Stellmaker<br>NCCTG *Research Base* Quality Control Specialist<br>Phone: (507) 538-0268<br>Fax: (507) 266-7240<br>E-mail: stellmaker.paula@mayo.edu
	Drug administration, infusion pumps, nursing guidelines	Dianne M. Herrera, R.N.<br>NCCTG *Research Base* Nurse<br>Phone: (507) 284-2459<br>Wanda DeKrey, R.N.<br>NCCTG Member Nurse<br>Phone: (701) 780-6741
	Forms completion and submission	Fern G. Lowe<br>NCCTG Member Clinical Research Associate<br>Phone: (306) 655-2669
Add 2,6	Protocol document, consent form, Regulatory issues	Sara Braun<br>NCCTG *Research Base* Protocol Development Coordinator<br>Phone: (507) 538-8226<br>Fax: (507) 284-5280<br>E-mail: braun.sara@mayo.edu
Add 4,6,7	Pathology	Christine R. Maszk<br>NCCTG *Research Base* Pathology Coordinator<br>Phone : (507) 266-8919<br>Fax : (507) 284-9628<br>E-mail : maszk.christine@mayo.edu
	Adverse Events (AdEERS, MedWatch, Non-AER, AML/MDS)	Patricia G. McNamara<br>NCCTG *Research Base* SAE Coordinator<br>Phone: 507/266-3028<br>Fax: 507/284-9628<br>E-mail: mcnamara.patricia@mayo.edu
Add 4,7	Technical problems with electric form entry	Vicki Bryhn<br>NCCTG *Research Base* Data Management Specialist<br>Phone: (507) 266-5350<br>Fax: (507) 538-0906<br>E-mail: bryhn.vicki@mayo.edu
Add 6,7	Non-paraffin biospecimens	Jacqueline M Lafky<br>NCCTG *Research Base* Biospecimen Resource Manager<br>Phone: (507) 538-0602<br>Fax: (507) 266-0824<br>Email: lafky.jacqueline@mayo.edu

***** No waivers of eligibility per NCI

Index

Schema

1.0 Background

2.0 Goals

3.0 Patient Eligibility

4.0 Test Schedule

5.0 Stratification Factors

6.0 Registration/Randomization Procedures

7.0 Protocol Treatment

8.0 Dosage Modification Based on Adverse Events

9.0 Ancillary Treatment

10.0 Adverse Event (AE) Reporting and Monitoring

11.0 Treatment Evaluation Using RECIST[53] Criteria

12.0 Descriptive Factors

13.0 Treatment/Follow-up Decision at Evaluation of Patient

Add 6 14.0 Translational/Pharmacological Studies

15.0 Drug Information

16.0 Statistical Considerations and Methodology

17.0 Pathology Considerations

18.0 Records and Data Collection Procedures

19.0 Budget

20.0 References

Add 2 Appendix IA - Consent Form – Cohort I

Add 2 Appendix IB - Consent Form – Cohort II

Appendix II - Patient Instruction Sheet

Add 4

Schema

NOTE: Cohort I accrual is complete as of Addendum 4. All new patients will be accrued to the Cohort II dose levels listed below from this point on.

Add 2

Cohort I*	Cohort II†
Registration (UGT1A1 genotyping may determine dose beyond cycle 1) (Add 2,4,6)	Registration (UGT1A1 genotyping results required prior to registration only if total bilirubin > upper limit of normal (ULN) but ≤ 2.0 x ULN)
↓	↓
At least 6 patients who carry the UGT1A1*28 polymorphism and at least 6 patients without the UGT1A1*28 polymorphism (Add 4,5)	28 additional patients enrolled (see Section 16.12),
↓	↓
Gemzar + CPT-11 Weekly x 4 Followed by 2-Week rest period (Add 4)	Gemzar + CPT-11 Weekly x 3 Followed by 1-Week rest period (cycle length = 4 weeks)
↓	↓
Unacceptable toxicity<br>Disease progression<br>Patient refusal	Unacceptable toxicity<br>Disease progression<br>Patient refusal
↓	↓
Discontinue Treatment	Discontinue Treatment
↓	↓
Event Monitoring<br>Follow-up per Section 18.0<br>**(As of Addendum 8, patients no longer need to be followed)** (Add 2,8)	Event Monitoring<br>Follow-up per Section 18.0<br>**(As of Addendum 8, patients no longer need to be followed)**

Cohort I: Starting dose (Level 0): GEMZAR 1000(mg/m^2), CPT-11 75 (mg/m^2)

Add 4 Cohort II: Starting dose (Level 0): GEMZAR 750 (mg/m^2); CPT-11 (75 mg/m^2)

Add 4 Cohort I Cycle = 6 weeks

Add 4 Cohort II: Cycle = 4 weeks

Add 3

Generic name: Gemcitabine<br>Brand name: Gemzar®<br>Mayo abbreviation: GEMZAR<br>Availability: Eli Lilly and Company	Generic name: Irinotecan<br>Brand name: Camptosar®<br>Mayo abbreviation: CPT-11<br>Availability: Commercial

Add 2

- *In Cohort I, at least 6 patients who carry the UGT1A1*28 polymorphism (either 6/7 or 7/7 promoter TA repeats) and at least 6 patients without the UGT1A1*28 polymorphism (6/6 TA repeats) will be enrolled and treated at the starting dose level 0 = GEMZAR 1000 mg/m^2, CPT-11 75 mg/m^2. Accrual will be suspended and Cohort I patients (at least 12) will be observed for a minimum of 6 weeks for adverse events before any additional patients will be entered.

Add 4

- †All potentially eligible patients will be considered for Cohort II. However, baseline total bilirubin will be used along with UGT1A1 genotype to determine eligibility (see section 3.16).

1.0 Background

1.1 Carcinoma of unknown primary (CUP) accounts for about 5% of all cancer diagnoses. Despite an often extensive search, the primary site of disease is not usually discovered. The most common sites found at autopsy series include lung and GI sites (pancreas, colon, stomach, and liver).

1.11 In the work-up of a patient with an unknown primary, it is important to determine if the patient falls into one of the more treatable subcategories. These include:

- women with axillary adenopathy, suggesting breast CA primary.
- women with peritoneal carcinomatosis, suggesting ovarian/primary peritoneal CA.
- men with prominent blastic bony metastasis, suggesting prostate CA.
- squamous cell CA of the neck nodes/inguinal nodes.
- younger men with poorly differentiated mediastinal or retroperitoneal mass, suggestive of germ cell origin.
- carcinoma with neuroendocrine features

Immunostains can be helpful in the diagnosis when H&E is not clear. These should be performed to rule out subsets of malignancies that are treated in a different manner (1). These include:

- Keratin or epithelial membrane antigen (positive in carcinoma)
- S-100 or HMB45 (melanoma markers)
- LCA (CD45) (hematolymphoid)
- Chromogranin or synaptophysin (neuroendocrine)

1.12 Patients with well-differentiated carcinomas that do not fit into these groups have a poor prognosis. Median survival is usually <8 months. Patients with poorly differentiated carcinoma (PDC) also have a poor prognosis, but may respond better to CDDP-based chemotherapy (1,2). Single agents have been evaluated in patients with unknown primaries, including 5FU, CDDP, oral etoposide, and mitomycin, with response rates of 10-20%. In general, CDDP and 5FU-based regimens have shown response rates of 20-30%, with median survival of 5-8 months (1,2). Newer, broad-spectrum combinations that include Taxol have yielded even better results (3). Parvadis et al conducted a phase II study using Taxol/CDDP, reporting a response rate of 41% with median survival not yet reached (>8mo) (4).

1.2 Recently, combination chemotherapy using paclitaxel, VP-16 and CDDP has shown promising activity in both PDC and well-differentiated carcinoma of unknown primary. Greco and Hainsworth treated 55 patients with CUP in a phase II trial using CBDCA 6AUC, Taxol 200 mg/m^2, and VP16 50/100 mg, d 1-10. They reported overall RR 47%, with 13% CR. Activity was seen in all subgroups treated. Median survival was 13.4 months. Toxicity was mainly hematologic, with 56% grade 3 leukopenia and 20% grade 4 leukopenia (3).

1.3 Two drugs that also have a broad spectrum of antineoplastic activity include gemcitabine and irinotecan. Gemcitabine is a fluoridated pyrimidine that inhibits DNA and RNA synthesis and repair (5). Gemcitabine has been used extensively in the treatment of lung and pancreatic cancer, and also shows activity against stomach, breast and ovarian cancers. Gemcitabine is well tolerated, with mild myelosuppression and minimal non-hematologic toxicity (6-11). It has been shown to improve tumor-related symptoms in patients with NSCLC and improves QOL in patients with pancreatic cancer (8-11). Gemcitabine has also been used as a single agent in treating patients with unknown primary carcinomas who had progressed on first line treatment. Response rates were 8%, with 25% of patients having stable disease (12). In a recent report, Greco et al. reported a new combination of gemcitabine, Taxol, and carboplatin for patients with unknown primary cancer. The response rate with the three-drug regimen was 25%, with median survival of 9 months, which is similar to other commonly used regimens. In this study, responding or stable patients continued to receive weekly Taxol, but did not benefit further from this additional treatment. The most common side effects with this regimen were myelosuppression, with grade 3/4 leucopenia in 46%, and grade 3/4 thrombocytopenia in 43% (13).

1.31 Irinotecan is a camptothecan analog. Its active metabolite, SN-38, inhibits topoisomerase I by stabilizing the enzyme-DNA complex. This leads to a DNA stand break and inhibition of replication (14, 15). Irinotecan has good activity in both gastrointestinal malignancies as well as lung cancer. Response rates in NSCLC range from 15-34%, with median survivals 6.2-9.8 months (16-19). Studies have also shown activity in breast, ovarian, cervical, SCLC, and pancreatic cancer (20). The main toxicities of irinotecan are diarrhea and neutropenia (16-20).

1.32 There is preclinical evidence that the combination of gemcitabine and irinotecan are synergistic (21, 22). Bahadori et al. conducted experiments using a small-cell lung cancer cell line, and found the combination to be synergistic. They postulate that if gemcitabine is available at the time of a DNA strand break, G triphosphate can be inserted into DNA, leading to irrepairable damage and cell death. CPT-11, a topoisomerase I inhibitor, causes DNA strand breaks, and could facilitate this incorporation of G triphosphate, leading to synergistic effect of the combination (22). Studies looking at dose and schedule-dependent effects of this combination of drugs are underway. At Mayo Clinic, the BxPC-3 hu pancreatic CA cell line showed synergistic activity with the sequence of gemcitabine followed by SN-38 (personal communication - S. Kaufmann). Given the extremely broad spectrum of antineoplastic activity, different mechanisms of action, and potential for synergism, the combination of these two drugs seems ideal for the empiric treatment of carcinoma of unknown primary.

1.33 Three phase I studies have combined these drugs in different schedules.

1.331 The first study treated 19 patients with solid tumors (refractory to standard therapy, or for which no standard therapy is available) (23). Gemcitabine was given at 1000mg/m^2 over 30 min, d1 and d8 Q3wks. CPT-11 was given in increasing doses (50,75,100,115m /m^2) over 90min, after gemcitabine, d1 and d8, Q 3 weeks. Dose-limiting toxicity (DLT) was diarrhea, which was grade 3 or 4 in 3/19 patients at a dose level of

CPT-11 100-115 mg/m^2. Cholinergic toxicity was noted in 2 patients at 115 mg/m^2. Hematologic toxicity was not dose dependent (maybe due to day 8 dose reductions) and cumulative toxicity was only observed in two patients at the highest dose level. Grade 2 and 3 neutropenia occurred in 4 patients at doses of 100-115mg/m^2. Grade 2-3 thrombocytopenia occurred in only 2 patients at 100-115mg/m^2. Non-hematologic toxicity was mild, and consisted of the following:

- Nausea grade 3: 3/19
- Vomiting grade 3: 1/19
- Diarrhea grade 3 or 4: 3/19

1.332 The authors concluded that the maximum tolerated dose (MTD) of irinotecan, when given with fixed dose gemcitabine (1g/m^2) was 100 mg/m^2. At the MTD, one of five patients was unable to receive day 8 therapy as a result of grade 3 thrombocytopenia. Eighteen patients were evaluable for response; three had PR's. Two of these patients had pancreatic CA and one had unknown primary adenocarcinoma. This latter response duration was 15 months.

1.333 O'Reilly also reported a phase I study with fixed dose gemcitabine (1g/m^2) and irinotecan (45, 60, 80, 100 mg/m^2) given on d 1, 8 and 15, Q 4 weeks (24). Arm A received gemcitabine followed by CPT-11, and Arm B received CPT-11 followed by gemcitabine. Grade 3 diarrhea and fatigue were DLT. MTD was found to be CPT-11 at 60mg/m^2 for arm A. Arm B is still accruing. So far, there were no differences in pharmacokinetics between either arm of the study, but hematologic toxicity appears greater with Arm B. There was one response in a patient with gastric cancer. Fourteen patients had stable disease.

1.334 A recently completed Mayo Clinic phase I study also used the combination of escalating dose gemcitabine (600, 800, 1000 mg/m^2) followed by irinotecan (75, 100, 125 mg/m^2) weekly for 4 weeks out of six weeks (25). Twenty-four patients were enrolled. No major responses were seen, but patients were heavily pre-treated. Dose limiting toxicity (DLT) was hematologic, and occurred at doses of gemcitabine 1000 mg/m^2 and irinotecan 125 mg/m^2. At the maximum tolerated dose (gemcitabine 1000 mg/m^2, irinotecan 100 mg/m^2) 1/6 patients completed 2/4 weeks of treatment, and three other patients completed 3/4 weeks. Of the remaining patients, one required a dose reduction but both received all four weeks of treatment. Most of these missed days were due to hematologic toxicity. At the MTD, two patients had grade 3 neutropenia, and grade 3/4 thrombocytopenia occurred in 17%. Diarrhea was the most common non-hematologic toxicity. At the MTD, three patients had grade 1 diarrhea, and one patient had grade 4 diarrhea. Other mild toxicities included anorexia (12 patients), alopecia (9 patients), fatigue, nausea, vomiting, and weight loss.

1.34 Two phase II studies have been reported in preliminary form.

1.341 Roca Lima et al., reported a phase II trial of first line irinotecan and gemcitabine in patients with advanced pancreatic cancer (26). Forty-five patients were treated in 8 sites. Doses were gemcitabine 1000mg/m^2, irinotecan 100mg/m^2, d1,8 every 3 weeks. Treatment was well tolerated, with full doses of irinotecan and gemcitabine given in 91% and 88%. Grade 3 diarrhea occurred in 6.7%, grade 3 or 4 neutropenia in 15.5%, and grade 3 or 4 thrombocytopenia in 8.9%. Response rate was 20%, decrease in CA19-9 by 50% occurred in 32.5%, median TTP was 2.9 month, and median survival 6 months.

1.342 The second phase II study was also in patients with advanced pancreatic cancer (27). Twenty-one chemo-naïve patients were treated with gemcitabine 900 mg/m^2 d1, 8 and irinotecan 300 mg/m^2 on d8, Q 3 weeks. Grade 3 or 4 neutropenia was seen in 36%, with 4 neutropenic fevers (one death). Grade 3 or 4 thrombocytopenia was seen in 8%. Grade 3 diarrhea occurred in 1 patient, and grade 2 or 3 asthenia was observed in 43%. Twenty patients were evaluable for response. PR was seen in 15% (3pts), stable disease in 40% (8 pts). Median TTP was 30 weeks, and median survival not yet reached.

1.35 As technology improves, and more discoveries are made about the genetic and molecular nature of cancer, it is likely that many cases of unknown primary cancer will be defined on the basis of immunohistochemistry and molecular techniques. This may direct treatment with a particular regimen. Currently, the immunohistochemical stains are useful to categorize patients into broad groups, such as hematologic, carcinoma, melanoma, sarcoma, etc. but have yet been refined enough to pinpoint the actual organ of origin. Immunostains such as CK7/20 have been used in the research setting, but are not widely clinically used, and are not specific for diagnosis (28). However, certain patterns of staining are characteristic of a given primary source, and may be useful to direct therapy. The translational component of this study proposes to stain all specimens for CK7/20 in an attempt to ascertain which patterns may be predictive of response to this regimen. This will be an exploratory analysis, as the numbers in this phase II study are too small to make any firm conclusions. We will also request the tissue blocks be retained here so that we may pursue additional translational studies in the future in conjunction with our pathology colleagues.

1.36 Irinotecan and gemcitabine, as shown in the above studies, is potentially active and tolerable when given in combination. Given the broad spectrum anti-neoplastic activity of these two agents, with well-known activity in both lung and GI malignancies, we propose a phase II trial using this combination in patients with carcinoma of unknown primary.

1.4 Response to treatment, or lack of, is dependent upon a variety of factors. Potentially important factors include sensitivity to the agent, achieving appropriate levels of the agent, and avoiding unacceptable toxicity. The latter two factors are influenced by the uptake, metabolism, and distribution of the agent. Individual variations in drug metabolism may greatly influence the efficacy and tolerability of therapy. However, the

ability to measure or predict individual variations in drug metabolism is quite limited. The evolving field of pharmacogenetics is helping to define factors that may contribute to individual variations by assessing factors such as genetic polymorphisms. As a better understanding of individual variation in response to therapy is obtained it may be possible to being tailoring therapy for individual patients.

1.41 The results of irinotecan clinical trials and *in vitro* work now suggest that the major dose limiting toxicities of irinotecan, diarrhea and myelosuppression may be genetically determined. Irinotecan, a semisynthetic derivative of camptothecin, is converted *in vivo* to SN-38, which is 1000 times more potent of an inhibitor of topoisomerase I (29-30). SN-38 covalently stabilizes the enzyme-DNA complexes, (32) resulting in strand breaks and subsequent cytotoxicity (33-36). SN-38, is inactivated by the polymorphic hepatic uridine diphosphate glucuronosyl-transferase 1A1 (UGT1A1) enzyme (37). A dinucleotide repeat polymorphism in the TATA sequence of the promoter region of UGT1A1, leads to significantly lower SN-38 glucuronidation rates in liver samples heterozygous or homozygous for the $(TA)_7TAA$ polymorphism (38). In a retrospective study, the presence of the UGT1A1*28 polymorphism was associated with a 7-fold increased risk for severe toxicity (leukopenia/diarrhea) when compared to patients without the *28 polymorphism (39). The first prospective phase I pharmacogenetic study of CPT-11 was recently reported (40). In this study, patients receiving irinotecan were noted to have significantly lower SN-38 glucuronidation rates than those without the *28 polymorphism. Furthermore, severe toxicity (grade 3/4 diarrhea or neutropenia), was seen only in those patients with the *28 polymorphism.

The first large prospective study reporting the association between the UGT1A1 *28 polymorphism and CPT-11 related toxicity and response was recently presented (41). This data is derived from a large prospective NCCTG study (N9741) in which patients with metastatic colorectal cancer were randomized to one of 3 different arms. In 2 of those arms, patients received either CPT-11, 5-FU, and leucovorin, or CPT-11 + oxaliplatin. The findings from this study showed that patients who carried the *28 polymorphism had significantly higher rates of grade 4/5 neutropenia (35% for 7/7 variant, 16% for the 6/7 variant) compared with patients with the 6/6 variant (8%). Perhaps even more importantly, response rates were significantly different, as patients who carried the 6/6 variant had significantly decreased response rates compared with those patients who carried the *28 variant.

Based on the accumulated evidence, it is clear that one dose (based on body surface area) does not fit all for patients receiving CPT-11. Therefore, stratifying by UGT1A1 genotyping is one way in which CPT-11 induced toxicity may be reduced.

Based on the Mayo Clinic phase I study of gemcitabine and CPT-11 (42), the MTD of gemcitabine was 1000 mg/m^2 weekly and CPT-11 (100 mg/m^2) weekly. However, at this dose level only 1/6 patients were able to complete all four weeks of treatment. Of the remaining 5 patients, 3 completed 3/4 weeks, 1 completed 2/4 weeks and the last patient required dose reductions in order to complete all 4 weeks of treatment. Toxicities accounting for missed weeks of

treatment or does reductions were predictable and included hematologic toxicity (4 patients) and diarrhea (1 patient).

Based on these results, it is unclear whether those patients who carry the UGT1A1 *28 polymorphism, will be able to tolerate a dose of weekly gemcitabine (1000 mg/m2) and CPT-11 (100 mg/m2). Furthermore, those patients who do not carry this polymorphism (6/6 repeats) may receive subtherapeutic doses of CPT-11 if they are dosed in the same manner as those with the *28 polymorphism.

Add 2

Therefore, in this study, we will enroll patients to 2 different cohorts, and all patients will be prospectively genotyped for UGT1A1. For Cohort I, we will begin by enrolling the minimum number of patients required such that at least 6 evaluable patients carry the UGT1A1*28 polymorphism, and at least 6 evaluable patients do not carry it. These Cohort I patients will receive gemcitabine at dose level = 1000 mg/m^2, and CPT-11 at dose level = 75 mg/m^2 (dose level 0). Note that the dose of CPT-11 is 25 mg/m^2 lower than the MTD of CPT-11 reported on the Mayo Clinic phase I study. (42) Following the enrollment of the Cohort I patients (i.e., at least 12), accrual will be suspended and the first 6 evaluable patients from each polymorphism group will be observed for a minimum of 6 weeks for adverse events before any additional patients will be entered. For Cohort II, a maximum of 37 patients (see Section 16.14 for details) will be prospectively genotyped and doses will be determined and subsequently administered based on the results of the feasibility portion (Cohort I portion) of this study.

In addition to the UGT1A1*28 polymorphism, we will also explore the impact of the recently described common variations (-3279G>T and –3156G>A) within the UGT1A1 phenobarbital-responsive enhancer module (43, 44). The –3279 variant has been associated with a significant (35%) increase in bilirubinemia in Asian patients with Gilbert's syndrome (43). Furthermore, because both the –3279 and the -3156 variants are in linkage disequilibrium with the $(TA)_n$ polymorphism (*28), common haplotypes comprising these variants may impact UGT1A1 glucuronidation (44). The haplotypes comprising –3279T and TA_6 (0.53) and –3279G and TA_7 (0.31) are most common in Caucasians where the presence of the TA_7 allele is known to impact SN-38 glucuronidation (40, 45). In addition, the haplotype comprising the –3279G and TA_6 variants (q=0.07 in Caucasians) may also impact SN-38 glucuronidation compared with the common –3279T and TA_6 haplotype. Accordingly, we will develop assays to detect both the –3279 and-3156 variants and explore whether haplotypes which comprise these variants impact toxicity and outcome in this phase II trial.

Finally, common allelic variants in exons 12, 21, and 26 of the *MDR1* gene are in linkage disequilibrium and may be associated with MDR1 expression and clinical outcome (46). Because P-glycoprotein plays an important role in the biliary excretion of CPT-11, MDR1 gene polymorphisms may impact CPT-11 pharmacokinetics (47). Indeed, a recent study found that the MDR1 1236 C>T variant was associated with a significant increase in CPT-11 and SN-38 exposure (48). Accordingly, we will explore whether the common polymorphisms in exons 12, 21, and 26 are associated with the clinical outcomes of response and toxicity in this phase II study.

1.42 The pharmacogenetics of gemcitabine is not well understood. With the current investigational approaches now available to better define individual variations in response to therapy, we are in a unique position to explore these variations in this trial. We plan to obtain appropriate blood samples for pharmacogenetic studies with gemcitabine. For gemcitabine, the laboratories of Drs Weinshilboum and Ames are currently involved in resequencing studies with several nucleoside transporters, cytidine deaminase, dCMP deaminase, deoxycytidine kinase, and deoxycytodilate kinase. Upon characterization of functional polymorphisms in these genes, we will be prepared to genotype for these mutations.

Add 4

1.5 As of Addendum 4, Cohort I is closed to accrual. All new patients will be accrued to Cohort II. Patients accrued to Cohort I on or after the Addendum 4 effective date will be added to Cohort II as noted in Section 16.1; therefore total sample size will be 42 patients (i.e., previously accrued Cohort I patients
[=14 patients] plus Cohort II patients [=28 patients]).

Add 6

1.6 Background for 92 gene RT-PCR cancer classification assay. The availability of technology allowing the study and comparison of gene expression signatures provides the opportunity to base the classification of human malignancies on a more objective and comprehensive set of data than allowed by the presently used clinical pathology criteria, that even when dealing with a known primary cancer site are hindered by a degree of subjectivity of interpretation.(63)

AviaraDx has developed a technology which enables the classification of 39 cancer types (64). This is a molecular-based methodology that quantifies the expression of 92 genes within a tissue cancer biopsy via real time RT-PCR. The clinical utility of this technology is the determination of the primary anatomical origin of a metastatic cancer.

A full description of this is found in the Ma et al (64). In brief terms, the development of the 92-gene assay was completed in five major steps. First, a 22,000 gene microarray was used to build a gene expression dataset of 578 cancer samples representing 39 tumor types. Second, 1001 genes were selected *in-silico* from this database based on their varying degrees of tumor-specific expression profiles. This was completed by dividing up the dataset into a training and test set and selecting the top 30 genes with the highest ability to discriminate at a specific node within the tumor taxonomy hierarchy of known cancer classification (e.g., a node for epithelial versus non-epithelial cancers). Thirdly, these 1001 genes were further narrowed down *in-silico* by the use of genetic algorithms to a non-redundant set of 126 genes that represented the overlap of the most optimal set of genes (74 genes) plus those genes most frequently found in the 100-derived genetic algorithm gene sets (90 genes). Fourth, real-time RT-PCR assays were developed for the 126 genes and this was further narrowed to the 87 genes whose individual analytical performance re-capitulate the expression patterns originally observed within the microarray-based dataset. Finally, a set of 5 genes were identified to enable the normalization of data from varying amounts of sample input (i.e., RNA) into the assay. In total, the 92-gene assay contains 87 genes for tumor classification and 5 genes for sample input normalization.

Once the 92-gene assay was developed, it was then used to generate a dataset consisting of the gene expression profiles of 87 genes for 578 cancer samples representing 39 cancer types. To classify a sample of unknown class, we first calculated its distance d to each

instance in the training set as the 1- Pearson correlation coefficient. The top k nearest neighbors were then examined for their class labels, and the unknown case was assigned to the class with the largest summed weight (1/d). The overall success rate for classifying 39 cancer types was 88% (64).

2.0 Goals

2.1 Primary goals:

2.11 To evaluate the response rate of the combination of irinotecan and gemcitabine in patients with unknown primary cancer.

2.12 For Cohort I patients, to assess the adverse event profile and tolerability of the combination of gemcitabine and irinotecan in patients with unknown primary carcinoma, based upon the presence or absence of the UGT1A1 *28 polymorphism.

Add 4

2.13 For Cohort II patients, to assess the adverse event profile and tolerability of the combination of gemcitabine and CPT-11, based on the interval dose reductions and schedule change.

2.2 Secondary goals:

2.21 To assess time to progression (TTP) and overall survival (OS).

Add 5

2.22 To perform immunohistochemical studies using CK7/20 to determine if certain patterns of staining are associated with response to therapy; we will also store tissue blocks at the NCCTG Tissue Repository for future translational studies.

2.23 To assess whether variation in multiple different genes whose protein products are involved in the uptake, metabolism, and distribution of Gemzar and CPT-11 affect clinical outcomes (response, toxicity).

Add 4

2.24 To determine whether cellular measurements of dFdCTP reflect the aggregate consequences of cellular uptake, anabolism and catabolism of gemcitabine by measuring intracellular triphosphates in the mononuclear cells. This pharmacologic or intervening phenotype will be used for correlation with genotypes determined in this study

Add 6

2.25 Determine primary origin of cancer of unknown primary (CUP) samples by completing a 92-gene RT-PCR cancer classification assay.

Add 6

2.26 Determine whether the 92-gene assay results are correlated with clinical response to CPT-11/Gemcitabine

3.0 Patient Eligibility

3.1 Required Characteristics

Add 1

3.11 Carcinoma of unknown primary after the following diagnostic procedures have been performed and are unrevealing of the primary site (see Sections 14.1 and 14.2):

- Complete history and physical
- CBC, chemistries
- UGT1A1 genotyping is required for all patients. However, results are required to determine eligibility only if total bilirubin > upper limit of normal (ULN) but ≤ 2.0 x ULN (see section 3.16) (Add 2,4,6)
- Chest x-ray and/or CT scan
- Abdominal CT
- Directed evaluation of symptomatic areas
- Mammogram in women
- Colonoscopy in patients with liver metastasis to exclude a colon primary

3.12 Biopsy proven carcinoma of undetermined origin with any of the following light microscopic diagnoses:

- Adenocarcinoma
- Poorly differentiated non-small cell carcinoma
- Poorly differentiated squamous carcinoma

Add 2

If the diagnosis of the above histologies is not clear by H&E, in the opinion of the home pathologist, the following stains are required to rule out certain subsets of malignancies:

- Keratin or epithelial membrane antigen (positive)
- S-100 or HMB45 (melanoma markers) (negative)
- LCA (CD45) (hematolymphoid) (negative)
- Chromogranin or synaptophysin (negative)
- Thyroid Transcription Factor 1 (negative) (Add 2)

If these stains cannot be performed at the treating institution, biopsy blocks should be sent to the NCCTG Pathology Coordinator for testing before patients can enter study (see Section 17.1).

Add 2,5

3.13 Patients must have measurable disease as defined in Section 11.0. For patients having only lesions measuring at least 1 cm to less than 2 cm must use spiral CT imaging for both pre- and post-treatment tumor assessments.

Add 2

3.14 ECOG performance status (PS) 0, 1, or 2.

Add 2

3.15 Age ≥18 years.

3.16 The following laboratory values obtained ≤14 days prior to registration:
- PLT ≥ 100,000
- Granulocytes ≥1500
- Total bilirubin:

Add 4,6

If ≤ ULN: Patient is eligible and UGT1A1 genotyping results are not required prior to registration (DNA sample is still required)

If > ULN but ≤ 2.0 x ULN: UGT1A1 genotyping results must be available prior to registration. Eligibility is based on the following genotype results:
  - UGT1A1 6/6: not eligible
  - UGT1A1 6/7 or 7/7 patients: eligible
- AST ≤3x ULN (unless liver mets, then ≤5 x ULN)
- Alk Phos ≤3x ULN
- Creatinine ≤2.0 x ULN

3.17 Life expectancy ≥12 weeks.

Add 4

3.18 Willingness to provide blood and tissue samples for research testing.

3.2 Contraindications

3.21 Any of the following subsets of patients:
- Neuroendocrine tumors
- Women with axillary nodes only
- Women with adenocarcinoma of peritoneum
- Carcinoma involving only one site, with resectable tumor at that site
- Squamous carcinoma limited to cervical, supraclavicular, or inguinal lymph nodes
- Men with poorly differentiated mediastinal or retroperitoneal tumor with stains suggestive of germ cell origin or serum tumor markers (AFP/HCG). These tumor markers are only required if the patient fits this clinical scenario.
- Men with prominent blastic bony metastasis or markedly elevated PSA, suggesting prostate origin
- ≤4 weeks from major surgery

Add 2

- If immunostains are performed, and any of the below tests are positive:
  - Hematologic CD45+ (others such as CD2, CD20, CD30, CD43 also suggest hematologic origin)
  - Melanoma (S-100 or HMB45)
  - Chromogranin or synaptophysin

Add 2

  - Lung or thyroid origin (Thyroid Transcription Factor [TTF-1])

3.22 Prior chemotherapy.

3.23 Any of the following: (This study involves an investigational agent whose genotoxic, mutagenic, and teratogenic effects on the developing fetus and newborn are unknown.)
- Pregnant women
- Nursing women
- Men or women of childbearing potential who are unwilling to employ adequate contraception (condoms, diaphragm, birth control pills, injections, intrauterine device [IUD], surgical sterilization, abstinence, etc.)

3.24 Prior RT to >25% bone marrow.

3.25 Uncontrolled infection.

3.26 Brain or meningeal involvement.

Add 4 3.27 Any other severe concurrent disease which, in the judgment of the investigator, would make the patient inappropriate for entry into this study.

Add 4 3.28 History of invasive malignancy ≤5 years.

4.0 Test Schedule

Add 5 · Add 2,4 · Add 4 · Add 1,2,4

Tests and procedures	Prior to study entry	≤14 days prior to registration	Active-Monitoring Phase: Cycle 1, day 1	Active-Monitoring Phase: During treatment and intervals between treatment	Active-Monitoring Phase: Prior to subsequent cycles
Pathology review **ONLY** for institutions that cannot perform the stains needed to clarify histology (see Section 3.12)	X[8]				
History and exam, wt, PS		X			X
Height		X			
Tumor Measurement		X[9]			X[1]
Hematology group<br>ANC<br>HgB<br>WBC<br>PLT		X		Weekly	X
Chemistry group<br>(AST, Alk phos, creat, d. and T. bili, Ca,<br>Na, K, glucose)		X			X[2]
PT (INR)		X[3]		Weekly[3]	X[3]
Mammogram (women only)		X[4]			
CT chest		X[4]			X[7]
CT abdomen		X[4]			
Colonoscopy		X[5]			
Research blood sample[R]	X[10] (gemcitabine pharmacogenetics)		X[10] (Prior to gemcitabine infusion, at end of infusion, and 60 minutes following infusion)		
UGT1A1 genotyping	X[11]				
Tissue sample[R]	X[12]				
Serum or urine pregnancy test		X[6]			

1. After 2 cycles. Then, measurable lesions may be evaluated with alternate cycles.
2. Ca, glucose required only as clinically indicated.
3. For patients receiving concomitant warfarin.
4. ≤30 days prior to registration.
5. Colonoscopy to be performed in patients with liver metastasis or strong suspicion of a colon primary; ≤30 days prior to registration.
6. For women of childbearing potential only. Must be done ≤7 days prior to registration.
7. As clinically indicated.
8. Biopsy blocks should be sent to NCCTG Pathology Coordinator for testing before patients can enter study (see Section 17.1). If the institution is unable to provide a block, submit 15 regular charged slides (5 microns). (Add 2,4)
9. Imaging studies ≤30 days, physical evaluation ≤14 days. (Note: This is mandatory)
10. Note: Submission of blood samples is mandatory (gemcitabine genotyping and phenotyping studies). Kits are provided and must be used. See Section 14.0 (1,2,4)
11. Note: This test is mandatory: Blood will be collected and sent by overnight express. Once blood is received in the laboratory of Dr. Ames, results will be reported within 3 business days. See Section 14.0. Results of UGT1A1 genotyping are necessary only if T bili is > ULN but ≤2.0 x ULN (Add 2,4,6)

12 Tissue samples for research testing are mandatory (see Section 14.34).

R. Research funded. (Add 4)

5.0 Grouping Factor: Cohort: I (closed per Addendum 4) vs. II.

6.0 Registration/Randomization Procedures

Add 2,5, Update 1

6.1 To register a patient, call (507/284-4130) or fax (507/284–0885) a completed eligibility checklist to the Randomization Center between 8 a.m. and 4:30 p.m. central time Monday through Friday.

Add 2

6.2 IRB approval(s) is required for each treating site. A signed Cancer Trials Support Unit (CTSU) IRB Certification Form is to be on file at the CTSU Regulatory Office (fax 215-569-0206). This form can be found at the following Web site: www.ctsu.org/rss2_page.asp. Guidelines can be found under Quick Fact Sheets.

Add 2, Update 1

6.3 At the time of registration/randomization, Randomization Center personnel will verify the following:

- IRB approval at the registering institution
- Patient eligibility
- Existence of a signed consent form
- Existence of a sign authorization for use and disclosure of protected health information (USA institutions only)

At the time of registration/randomization, the following will be recorded:

- Patient has/has not given permission to store and use his/her blood sample(s) for future research of cancer.
- Patient has/has not given permission to store and use his/her tissue sample(s) for future research of cancer.

- Patient has/has not given permission to store and use his/her blood sample(s) for future research to learn, prevent, or treat other health problems.
- Patient has/has not given permission to store and use his/her tissue sample(s) for future research to learn, prevent, or treat other health problems.

- Patient has/has not given NCCTG permission to give his/her blood sample(s) to outside researchers.
- Patient has/has not given NCCTG permission to give his/her tissue sample(s) to outside researchers.

Add 2,4

6.4 Randomization Center will automatically register patients separately to the mandatory translational component of this study (see Section 14.0).

Add 2

6.5 Treatment on this protocol must commence at the accruing membership under the supervision of a NCCTG member physician.

Add 2

6.6 Treatment cannot begin prior to registration and must begin ≤7 days after registration.

Add 2

6.7 Pretreatment tests must be completed within the guidelines specified on the test schedule.

Add 2

6.8 All required baseline symptoms must be documented and graded on the on-study form.

Add 2

6.9a Study drug availability checked.

Add 5

6.9b Blood draw kit availability checked.

7.0 Protocol Treatment

Add 4 **7.1**

Treatment schedule for Cohort I-Use actual weight or estimated dry weight if fluid retention

NOTE: As of Addendum 4, all new patients will be accrued to the Cohort II at the dose levels listed below.

Agent	Dose	Route	Day	ReRx
GEMZAR	1000 mg/m^2	IV in 250/ml NS over 30 min	1, 8, 15, 22	Retreat q 42 days (6 weeks - includes a 2-wk rest period)
CPT-11	75 mg/m^2	IV in 500/ml D5W over 90 min, starting 30 min after completion of GEMZAR	1, 8, 15, 22	Retreat q 42 days (6 weeks - includes a 2-wk rest period)

Cycle = 6 weeks

Add 4 **Treatment schedule for Cohort II** -Use actual weight or estimated dry weight if fluid retention

Agent	Dose	Route	Day	ReRx
GEMZAR	750 mg/m^2	IV in 250/ml NS over 30 min	1, 8, 15	Retreat q 28 days includes a 1-wk rest period)
CPT-11	75 mg/m^2	IV in 500/ml D5W over 90 min, starting 30 min after completion of GEMZAR	1, 8, 15	Retreat q 28 days includes a 1-wk rest period)

Add 2.4

Cycle = 4 weeks

Add 2

7.2 Dose Adjustments (for Cohort I)

Add 2,4

NOTE: As of Addendum 4, all new patients will be accrued to the Cohort II at the dose levels listed below.

Levels	GEMZAR (mg/m^2)	CPT-11 (mg/m^2)
-2	750	35
-1	1000	50
**Starting dose (level 0)**	**1000**	**75**
+1*	1000	100

Add 2

Add 2 * If any patient in Cohort I without the UGT1A1*28 polymorphism (6/6 promoter TA repeats) completes a 6-week cycle of treatment at the starting dose (GEMZAR 1000 + CPT-11 75) without any missed or delayed doses and does not require any dose reductions, the dose may be escalated to dose level +1 with subsequent cycles (GEMZAR 1000 mg/m^2, CPT-11 100 mg/m^2). Further dose escalation for this Cohort is not permitted.

Add 2 7.3 Dose Adjustments (Cohort II)

Levels	GEMZAR (mg/m^2)	CPT-11 (mg/m^2)
-2	480	45
-1	600	60
**Starting dose (level 0)**	**750**	**75**

Add 2

Add 2,3,4 7.4 Cohort I: All patients accrued during the Cohort I phase will be treated at the starting dose level 0 = GEMZAR 1000 mg/m^2, CPT-11 75 mg/m^2. Only the first 6 evaluable patients in each group (i.e., first 6 evaluable patients who carry (are positive for) the UGT1A1*28 polymorphism and first 6 evaluable patients without (are negative for) the UGT1A1*28 polymorphism) will be used in the analysis of Cohort I patients (see 16.14). At the point in which at least 6 evaluable patients in each group have been enrolled, accrual will be suspended and Cohort I patients will be observed for a minimum of 6 weeks for adverse events before any additional patients will be entered. The starting dose for additional patients (referred to as Cohort II) will depend on the adverse events and missed number of doses seen in the first 12 evaluable (6 from each *28 polymorphism group) Cohort I patients.

Add 4 7.5 As of Addendum 4, a total of 11 Cohort I patients were evaluable for cycle 1 toxicity. For patients with the UGT1A1 6/6 genotype, a total of 6 patients were evaluable for cycle 1 toxicity. 3 patients developed at least grade 3 neutropenia and/or grade 3 thrombocytopenia, requiring dose omissions/delays. 1 additional patient developed grade 3 nausea/vomiting, diarrhea and grade 4 infection. For the UGT1A1 6/7 or 7/7 genotype, a total of 2/5 patients had grade 3 or 4 hematologic toxicity necessitating dose delays and an additional patient developed grade 3 neutropenia at the end of cycle 1 (not requiring dose reductions).

Add 4 This initial analysis demonstrates that grade 3 or 4 toxicity was not restricted to just the UGT1A1 *28 group, and that toxicity was seen broadly, regardless of genotype. Furthermore, the classic UGT1A1*28 related toxicity (which is grade 4 neutropenia), was seen in only 1/5 patients with the UGT1A1*28 genotype, suggesting that the dose of CPT-11 does not need to be modified for this group. Based on this, we will modify both the schedule of these drugs as well as the dose of Gemcitabine (but not CPT-11). These changes are reflected below.

Add 4 7.51 Cohort II: Changes to the Cohort II dosing and schedule are as follows:

1) Day 22 (week 4) will be dropped and the cycle will be reduced from 6 weeks to 4 weeks. Treatment will proceed weekly with both drugs for 3 weeks, followed by a 1 week rest period.

2) Gemcitabine will be reduced from 1000 mg/m^2 to 750 mg/m^2.

Additionally, we will perform an interim toxicity analysis and efficacy analysis once at least 6 patients from each genotype group have been accrued (UGT1A1 *28 versus UGT1A1 6/6). If the study meets the criteria for efficacy at the interim, additional recommendations for dose reductions or genotype-guided dosing will be based on the toxicity analysis at that time. (See section 16.0)

Add 4 7.6 Efficacy and toxicity analysis for Cohort II

An interim efficacy and toxicity analysis will be performed jointly once at least 13 patients have been enrolled to Cohort II and furthermore, at least 6 patients with and 6 patients without the *28 polymorphism (UGT1A1 6/7 and 7/7) have been enrolled (at least 13 and up to 15 patients). If the study does not meet the criteria for efficacy (see section 16), further accrual will cease to this study. If the trial does meet the criteria for efficacy, we will determine the frequency of toxicities in both UGT1A1 genotype groups. Furthermore, the overall cycle 1 toxicity rate for both genotype groups will be reviewed and a decision rendered regarding further dose reductions at that time (see Section 16.0 for details).

Add 4 7.7 Retreatment of patients: Decisions regarding retreatment of any patient will be based upon the type, severity, duration, and reversibility of the adverse event reactions. In general, patients who show improvement or stability of their disease will continue to receive treatment. Patients having objective progression of disease or clinical deterioration will not continue to receive treatment on this study. Patients who develop CNS metastasis will not continue to receive treatment on this study.

Add 4 7.8 During the tolerability assessment portion of the study, if a patient fails to complete the initial course of therapy for reasons other than adverse events, the patient will be regarded as treatment intolerant and will be replaced with an additional patient, to be treated at that same dose level. All adverse event information, however, will be used in the analysis.

8.0 Dosage Modification Based on Adverse Events - Strictly follow the modifications in this table for the first **two** cycles, until individual treatment tolerance can be ascertained. Thereafter, these modifications should be regarded as guidelines to produce mild-to-moderate, but not debilitating, adverse events. If multiple adverse events are seen, administer dose based on greatest reduction required for any single adverse event observed. Reductions apply to treatment given in the preceding cycle and are based on adverse events observed since the prior dose.

ALERT: *ADR reporting may be required for some adverse events (See Section 10)*

→ → Use Common Terminology Criteria for Adverse Events (CTCAE) v3.0 unless otherwise specified ← ←			
**CTCAE CATEGORY**	**ADVERSE EVENT**	**AGENT**	**DOSAGE CHANGE**
BASED ON WEEKLY ADVERSE EVENTS (NO CHEMO GIVEN DURING WEEKS 5 & 6 – REST WEEKS)			
Blood/Bone Marrow	Hematologic<br>PLT 50-75,000	GEMZAR<br>CPT-11	↓ dose level (see Section 7.2) for remaining weeks in cycle unless further dose reductions needed. If dose reductions pass dose level –2 for both drugs required, no further treatment should be given, and patient should go to event monitoring.
	ANC <1000 or<br>PLT <50,000		Omit dose for current week. Then ↓ one dose level (see Section 7.2) for remaining weeks in cycle if counts remain above ANC ≥1000/PLT ≥50,000. If adverse events persist for >2 weeks, patient goes off study and proceeds to event monitoring.
Nonhematologic other than diarrhea	Grade 3-4		Omit dose for current week. Then ↓ one dose level (see Section 7.2) for remaining weeks in cycle if adverse events resolve to ≤grade 2. If toxicities persist for >2 weeks, patient goes off study and proceeds to event monitoring.<br><br>In the case of nausea and vomiting, dose modification criteria should apply only when nausea and vomiting occur despite maximal anti-emetic therapy.
Pulmonary/<br>Upper Respiratory	Pneumonitis ≥Grade 2	GEMZAR	Discontinue treatment immediately and remove patient from protocol. Treatment with corticosteroids should be given according to established guidelines.
Coagulation[1]	Thrombotic microangiopathy (Hemolytic Uremic Syndrome) (HUS)		Discontinue treatment immediately
Gastrointestinal	Diarrhea<br>Grade 2-3	CPT-11	Omit dose for current week. Then ↓ one CPT-11 dose level (see Section 7.2) for remaining weeks in this cycle if adverse event resolves to <grade 2. Use reduced dose level for next cycle. If adverse events persist for >2 weeks, patient goes off study and proceeds to event monitoring.
	Grade 4		Omit dose for current week. Then ↓ two CPT-11 dose levels (see Section 7.2) for remaining weeks in this cycle if adverse event resolves to <grade 2. If adverse events persist for >2 weeks, patient goes off study and proceeds to event monitoring.

1. Recommended evaluation of suspected HUS: Evaluation should include CBC differential, Platelets, PT, PTT, fibrinogen, FDP (Fibrim degradation products), Anti thrombin 111, Von Willebrand factor, anti-nuclear antibody, rheumatoid factor, Compliment Cascade C3, C4, and CH_{50}, and anti-platelet antibodies, platelet-associated 1gG, and circulatin immune complexes. Renal evaluation should include creatinine, BUN, and urinalysis with microscopic examination. Other laboratory and hematological evaluations as appropriate should also be obtained, including peripheral bleed smear and free hemoglobin.

<table>
<tr><th colspan="4">AT TIME OF NEXT CYCLE (EVERY 6 WEEKS)</th></tr>
<tr><td colspan="4">BASED ON INTERVAL ADVERSE EVENTS</td></tr>
<tr><td colspan="4">If dose reductions were used in weeks 1-4 but treatment was not held, use the lowest dose level of drug from the past cycle to start this cycle.

If treatment was held for an adverse event in the last cycle, begin new cycle at one dose level below the last one given.</td></tr>
<tr><td colspan="4">BASED ON CURRENT PARAMETERS</td></tr>
<tr><td>Blood/Bone Marrow</td><td>Hematologic nadirs
ANC <1000
PL <100,000</td><td rowspan="2">GEMZAR
CPT-11</td><td rowspan="3">Hold Rx until counts >than these values then reRx based on above dose modifications. For nonhematologic toxicity, hold until ≤grade 1 and then reRx based on interval toxicity. If adverse events persist for >2 weeks, patient goes off study and proceeds to event monitoring.</td></tr>
<tr><td>Nonhematologic other than diarrhea</td><td>≥grade 3</td></tr>
<tr><td>Gastrointestinal</td><td>Diarrhea
Grade 1</td><td>CPT-11</td></tr>
</table>

9.0 Ancillary Treatment

9.1 All patients will be instructed to take Loperamide (Imodium or Kaopectate I-D) at the earliest sign of diarrhea (i.e., first poorly formed or loose stool, first episode of an increase from baseline of >2 bowel movements in one day) that occurs more than 12 hours after receiving CPT-11. Loperamide should be taken in the following manner: 4mg at the first onset of diarrhea, then 2 mg every 2 hours around the clock until diarrhea free for at least 12 hours. Patients may take loperamide 4 mg every 4 hours during the night. All patients will be given an instruction sheet (Appendix II) to take home on the first day of their treatment.

9.2 Atropine: Patients should remain in the treatment area for a minimum of one hour following completion of CPT-11 infusion for the first 2 infusions. Thereafter, the patients may leave after the completion of the infusion at their, and the investigator's, discretion. Diarrhea or abdominal cramping that occurs during, or within one hour after receiving CPT-11, should be treated with up to 1 mg of atropine IV (unless there is a relative or absolute contraindication to its use such as, but not limited to, closed angle glaucoma, urinary retention, or previous untoward reactions to the administration of atropine). Additional antidiarrheal measures should be used at the discretion of the treating physician.

9.3 Antiemetics: CPT-11 is moderately emetogenic and pretreatment with dexamethasone and ondansetron (or similar drug) should be used per standard protocols at each treating institution.

9.4 No other chemotherapeutic agents, biologic agents, or radiation may be used while the patient is on study.

9.5 Patients may receive all pre-study concomitant medications. If a patient is on warfarin, a prothrombin time (PT) should be monitored weekly. An increase in the PT has been reported in one patient receiving CPT-11.

9.6 Granulocyte Colony Stimulating Factor (G-CSF): Treatment with G-CSF may not be used to maintain a patient's blood counts for additional cycles of therapy.

10.0 Adverse Event (AE) Reporting and Monitoring

Add 4

10.1 This study will utilize the Common Terminology Criteria for Adverse Events (CTCAE) v3.0 for adverse event monitoring and reporting. The CTCAE v3.0 can be downloaded from the CTEP home page (http://ctep.info.nih.gov/CTC3/ctc_ind_term.htm). All appropriate treatment areas should have access to a copy of the CTCAE v3.0.

10.11 Adverse event monitoring and reporting is a routine part of every clinical trial. First, identify and grade the severity of the event using the CTCAE. Next, determine whether the event is expected or unexpected (refer to Section 10.12) and if the adverse event is related to the medical treatment or procedure (see Section 10.13). With this information, determine whether an adverse event should be reported as an expedited report (see Section 10.2). Important: All AEs reported via expedited mechanisms must also be reported via the routine data reporting mechanisms defined by the protocol (see Section 10.3 and 18.0).

Expedited adverse event reporting requires submission of an Adverse Event Expedited Reporting System (AdEERS) report(s). Other expedited reporting requirements and systems may also apply. Expedited and/or routine reports are to be completed within the timeframes and via the mechanisms specified in Sections 10.2 and 10.3. All expedited AE reports must also be sent to the local Institutional Review Board (IRB) according to local IRB's policies and procedures.

10.12 Expected vs. Unexpected

- The determination of whether an AE is expected is based on the agent-specific information provided in Section 15.0 of this protocol.
- Unexpected AEs are those not listed in the agent-specific information provided in Section 15.0 of this protocol.

10.13 Assessment of Attribution

When assessing whether an adverse event is related to a medical treatment or procedure, the following attribution categories are utilized:

Definite - The adverse event *is clearly related* to the agent(s).
Probable - The adverse event *is likely related* to the agent(s).
Possible - The adverse event *may be related* to the agent(s).
Unlikely - The adverse event *is doubtfully related* to the agent(s).
Unrelated - The adverse event *is clearly NOT related* to the agent(s).

10.2 Expedited Adverse Event Reporting Requirements

	Grade 4 or 5 Unexpected with Attribution of Possible, Probable, or Definite	Increased Incidence of an Expected AE[1]	Other Grade 4 or 5 ***or*** Any Hospitalization During Treatment[2]	Secondary AML/MDS[3]
Electronic FDA Form 3500 (MedWatch) to NCCTG within 5 days[4,6]	X	X		
Notification form: Grade 4 or 5 Non-AER Reportable Events/ Hospitalization Form to NCCTG within 5 working days[2,5]			X	
NCI/CTEP Secondary AML/MDS Report Form to NCCTG within 15 working days[5,6]				X

1. Any increased incidence of a known AE that has been reported in the package insert or the literature, including adverse event resulting from a drug overdose.
2. If the electronic FDA Form 3500 (MedWatch) has been completed, this form does not need to be completed. Any death more than 30 days after the patient's last study treatment or procedure which has an attribution of at least possibly treatment related must be reported as a Grade 5 AE, with CTCAE type
Add 4 and attribution assigned.
Add 3,4 3. Reporting for this AE required during or after treatment.
4. Submit electronically using the commercial pathway in the AdEERS system to the NCCTG SAE
Add 4 Coordinator. See the CTEP Home Page at http://ctep.cancer.gov for access to the AdEERS system.
5. Fax to the NCCTG SAE Coordinator, 200 First Street SW, Rochester, MN 55905, Fax 507-284-9628.
Add 3 6. The NCCTG SAE Coordinator will inform Eli Lilly and Company by fax of any serious adverse events experienced by a patient. Fax number is 317-277-0853.

All forms noted above are available for downloading from the NCCTG web site. Upon receipt of the above reports, the NCCTG Operations Office will forward reports to all regulatory agencies (including NCI, FDA, and others) as applicable. Specifically, and in accordance with the relevant section of the *NCI Guidelines: Expedited Reporting Requirements for NCI Investigational Agents,* the Operations Office will forward FDA Form 3500 (MedWatch) reports to the FDA and to NCI within the allotted (total) 10 working days and using one of the cited submission mechanisms.

Add 5 **Additional Instructions or Exceptions to AdEERS Expedited Reporting Requirements for Phase 2 and 3 Trials Utilizing an Agent Under a CTEP IND:**

- In the rare event when Internet connectivity is disrupted, a report may be prepared using the Adverse Event Expedited Report – Single Agent or Multiple Agents paper template (available on the CTEP Home Page at http://ctep.cancer.gov) and faxed to 301—230-0159.
  Refer to CTEP, NCI Guidelines: Adverse Event Reporting Requirements for back-up submission instructions. When internet connectivity is interrupted, a 24-hour notification is made to CTEP by telephone at 301-897-7479. Once internet connectivity is restored, an AE report submitted on a paper template or a 24-hour notification that is called in, must be entered into electronic AdEERS by the original submitter of the report at the site.
- Refer to Section 10.22 of this protocol for additional expedited reporting requirements.

10.22 Other Required Expedited Reporting

Add 5,6

EVENT TYPE	REPORTING PROCEDURE
Secondary AML/MDS	Reporting for this event required during and after completion of study treatment.<br>Submit the NCI/CTEP Secondary AML/MDS Report form within 15 days via remote data entry. The Operations Office will submit to NCI.
Other Grade 4 or 5 Events and/or Any Hospitalizations During Treatment Not Otherwise Warranting an Expedited Report	If an AdEERS report has been submitted, this form does not need to be submitted.<br>Enter into the remote data entry system within 5 working days of notification.

10.3 Adverse events to be graded at each evaluation and pretreatment symptoms/conditions to be evaluated at baseline per Common Toxicity grading unless otherwise stated:

	**Adverse Event/Symptoms**	**Baseline**	**Each evaluation**
# stools	# stools per day	X	
**CTC Category**			
Allergy/Immunology	Allergic reaction/hypersensitivity (including drug fever)	X	X
Constitutional Symptoms	Weight gain		X
	Weight loss		X
Dermatology/Skin	Rash/desquamation	X	X
Gastrointestinal	Anorexia	X	X
	Diarrhea		X
	Nausea	X	X
	Vomiting	X	X
Infection	Infection (documented clinically or microbiologically) with grade 3 or 4 neutrophils (ANC <1.0 x 10^9L) Select: -Abdomen NOS -Bronchus -Lung (pneumonia) -Bladder (urinary) -Urinary tract NOS	X	X
	Febrile neutropenia (fever of unknown origin without clinically or microbiologically documents infection) (ANC <1.0 x 10^9/L, fever ≥38.5°C)	X	X

10.31 Submit to the NCCTG Research Base via the NCCTG Case Report Form the following AEs experienced by a patient and not specified in Section 10.3:

Add 4

10.311 Grade 2 AEs deemed *possibly, probably, or definitely* related to the study treatment or procedure.

Add 4

10.312 Grade 3 and 4 AEs regardless of attribution to the study treatment or procedure.

Add 4

10.313 Grade 5 AEs (Deaths)

Add 4

10.3131 Any death within 30 days of the patient's last study treatment or procedure regardless of attribution to the study treatment or procedure.

Add 4 10.3132 Any death more than 30 days after the patient's last study treatment or procedure that is felt to be at least possibly treatment related must also be submitted as a Grade 5 AE, with a CTCAE type and attribution assigned.

Add 4 10.32 Refer to the instructions in the Forms Packet (or electronic data entry screens, as applicable) regarding the submission of late occurring AEs following completion of the Active Monitoring Phase (i.e., compliance with test schedule in Section 4.0).

11.0 Treatment Evaluation Using RECIST[53] Criteria

Add 4 11.1 Schedule of Evaluations: For the purposes of this study, patients should be reevaluated every 8 weeks (every 2 cycles) during Cohort II.

11.2 Definitions of Measurable and Non-Measurable Disease

Add 5 11.21 Measurable disease is defined as at least one lesion whose longest diameter can be accurately measured as ≥2.0 cm with conventional techniques or as ≥ 1.0 cm with spiral CT. Clinical lesions will only be considered measurable when they are superficial (e.g., skin nodules, palpable lymph nodes). Lesions on chest x-ray are acceptable as measurable lesions when they are clearly defined and surrounded by aerated lung. However, CT is preferable.

Add 5 11.22 All other lesions (or sites of disease), including small lesions (longest diameter <2.0 cm with conventional techniques or as < 1.0 cm with spiral CT) are considered non-measurable disease. Bone lesions leptomeningeal disease, ascites, pleural/pericardial effusions, lymphangitis cutis/pulmonis, inflammatory breast disease, abdominal masses (not followed by CR or MRI), and cystic lesions are all non-measurable.

11.3 Guidelines for Evaluation of Measurable Disease

Add 5 11.31 Measurement Methods:

- Add 5 All measurements should be recorded in metric notation (i.e., decimal fractions of centimeters) using a ruler or calipers.
- Add 5 The same method of assessment and the same technique must be used to characterize each identified and reported lesion at baseline and during follow-up. For patients having only lesions measuring at least 1 cm to less than 2 cm must use spiral CT imaging for both pre- and post-treatment tumor assessments.
- Add 5 Imaging-based evaluation is preferred to evaluation by clinical examination when both methods have been used at the same evaluation to assess the antitumor effect of a treatment.

Add 5 11.32 Acceptable imaging modalities for measurable disease: CT scan (conventional and spiral), MRI, chest x-ray, and physical examination.

- Conventional CT and MRI must be performed with cuts of 1.0 cm or less in slice thickness contiguously.

- Spiral CT must be performed using a 5 mm contiguous reconstruction algorithm. This specification applies to tumors of the chest, abdomen, and pelvis, while head and neck tumors and those of the extremities require specific procedures.

Add 5
- Ultrasound (US) is not acceptable to measure tumor lesions that are clinically not easily accessible.

Add 5
- Color Photography: In the case of skin lesions, documentation by color photography including a ruler to estimate the size of the lesion is recommended.

11.33 Measurement at Follow-up Evaluation:

- A subsequent scan must be obtained 4 weeks following initial documentation of an objective status of either complete response (CR) or partial response (PR).
- In the case of stable disease (SD), follow-up measurements must have met the SD criteria at least once after study entry at a minimum interval of 6 weeks (see Section 11.44).
- The cytological confirmation of the neoplastic origin of any effusion that appears or worsens during treatment when the measurable tumor has met criteria for response or stable disease is mandatory to differentiate between response or stable disease (an effusion may be a side effect of the treatment) and progressive disease.
- Cytologic and histologic techniques can be used to differentiate between PR and CR in rare cases (e.g., residual lesions in tumor types such as germ cell tumors, where known residual benign tumors can remain.)

11.4 Measurement of Effect

11.41 Target Lesions

All measurable lesions (as defined in Section 11.21) up to a maximum of 10 lesions representative of all involved organs should be identified as target lesions and recorded and measured at baseline. If the protocol specified studies are performed, and there are fewer than 10 lesions identified (as there often will be), there is no reason to perform additional studies beyond those specified in the protocol to discover new lesions. For any one organ, no more than 5 lesions need to be measured. Target lesions should be selected on the basis of their size (lesions with the longest diameter) and their suitability for accurate repetitive measurements (either by imaging techniques or clinically).

A sum of the longest diameter (LD) for all target lesions will be calculated and reported as the baseline sum LD. The baseline sum LD will be used as reference to further characterize the objective tumor response of the measurable dimension of the disease.

11.42 Non-Target Lesions

Add 5

All other lesions (or sites of disease) should be identified as non-target lesions and should also be recorded at baseline. Measurements are not required, and these lesions should be followed in accord with 11.433.

11.43 Response Criteria

11.431 All identified sites of disease must be followed on re-evaluation. Specifically, a change in objective status to either a PR or CR cannot be done without rechecking all identified sites (i.e., target and non-target lesions) of pre-existing disease.

11.432 Evaluation of Target Lesions

- Complete Response (CR): Disappearance of all target lesions.
- Partial Response (PR): At least a 30% decrease in the sum of the LD of target lesions taking as reference the baseline sum LD.
- Progression (PD): At least a 20% increase in the sum of LD of target lesions taking as reference the smallest sum LD recorded since the treatment started or the appearance of one or more new lesions.
- Stable Disease (SD): Neither sufficient shrinkage to qualify for PR nor sufficient increase to qualify for PD taking as references the smallest sum LD.

11.433 Evaluation of Non-Target Lesions

- Complete Response (CR): Disappearance of all non-target lesions.
- Stable Disease (SD): Persistence of one or more non-target lesions.
- Progression (PD): Appearance of one or more new lesions. Unequivocal progression of existing non-target lesions.

NOTE: Although a clear progression of “non-target” lesions only is exceptional, in such circumstances, the opinion of the treating physician will prevail, and the progression status will be confirmed at a later time by the study chair or a review panel.

11.44 Overall Objective Status

The overall objective status for an evaluation is determined by combining the patient’s status on target lesions, non-target lesions, and new disease as defined in the following table.

Target Lesions	Non-Target Lesions	New Lesions	Overall Objective Status
CR	CR	No	CR
CR	SD	No	PR
PR	Non-PD	No	PR
SD	Non-PD	No	SD
PD	Any	Yes or No	PD
Any	PD	Yes or No	PD
Any	Any	Yes	PD

11.45 Residual Disease: In some circumstances it may be difficult to distinguish residual disease from normal tissue. When the evaluation of complete response depends upon this determination, it is recommended that the residual lesion be investigated (fine needle aspirate/biopsy) before confirming the complete response status.

11.46 Symptomatic Deterioration: Patients with global deterioration of health status requiring discontinuation of treatment without objective evidence of disease progression at that time, and not either related to study treatment or other medical conditions, should be reported as PD due to "symptomatic deterioration." Every effort should be made to document the objective progression even after discontinuation of treatment due to symptomatic deterioration:

- Weight loss >10% of body weight.
- Worsening of tumor-related symptoms.
- Decline in performance status of >1 level on ECOG scale.

11.5 Formal statistical definitions of analysis variables involving response and disease progression are contained in Section 16.0.

12.0 Descriptive Factors

12.1 Predominant location of disease: Liver, lung, soft tissue, bone, other.

12.2 Pathology description: Histologic diagnosis and grade.

Add 2,4 12.3 UGT1A1*28 genotype: 7/7 TA repeats vs. 6/7 TA repeats vs. 6/6 TA repeats.

Add 2,4 12.4 UGT1A1*28 polymorphism group: Patients who carry the UGT1A1*28 polymorphism (either 7/7 or 6/7 TA repeats) vs. patients without the *28 polymorphism (6/6 TA repeats).

13.0 Treatment Follow-Up Decision at Evaluation of Patient

Add 2

13.1 Patients who are at least stable at the time of their reassessment and who have not experienced intolerable adverse events will continue with the protocol treatment at the same dose level until PD. The exception is for those patients in Cohort I who are negative for (do not carry) the UGT1A1*28 polymorphism (6/6 promoter TA repeats) who complete the first 6-week cycle of treatment at the starting dose (GEMZAR 1000 + CPT-11 75) without any missed or delayed doses and do not require any dose reductions. For these patients, the dose may be escalated to dose level +1 with subsequent cycles (GEMZAR 1000 mg/m^2, CPT-11 100 mg/m^2). Further dose escalation for this Cohort is not permitted.

Add 8

13.2 Those patients with disease progression, unacceptable adverse events, or refusal of further treatment will go onto the event-monitoring phase and will be followed every 3 months for a maximum of 2 years. **(As of Addendum 8, patients no longer need to be followed)**

Add 8

13.3 Patients who develop evidence of CNS disease will be taken off study and go to event monitoring. **(As of Addendum 8, patients no longer need to be followed)**

13.4 If a patient does not receive treatment and is classified as a cancel, it is not necessary to provide follow-up information. On-study material is to be submitted.

Add 6

14.0 Translational/Pharmacologic Studies: (See Section 1.4 for background)

14.1 Description of Assays

Add 2,4,5

NOTE: This blood sample is required for UGT1A1 *28 genotyping and for research testing. MDR genotyping, gemcitabine pharmacogenetics and gemcitabine phenotyping are mandatory. **Blood sample kits are available and must be used.**

Add 4

The laboratory utilizes allele-specific restriction fragment length polymorphism (RFLP) assays with GeneScan detection for detecting the presence of functional polymorphisms. The laboratory is continuously assessing and updating genotyping assay methodologies as well as adding assays for additional functional polymorphisms using the resources in Dr. Ames' and Dr. Weinshilboum's laboratories, the Mayo Cancer Center MicroArray Shared Resource and the Mayo Genomics Center Developmental Laboratory. An assay for the detection of the UGT1A1*28 promoter polymorphism (utilizing the method of Akaba et al (49) has been developed in the laboratory of Dr. Ames. Assays to detect both the UGT1A1 –3279 and –3156 variants have been developed. In addition, assays for the detections of functional polymorphisms in MDR1 exons 12, 21, and 26 are currently in place.

Add 4

Additionally, the collected DNA will be used for Gemcitabine pharmacogenetic studies. For gemcitabine, the laboratories of Drs Weinshilboum and Ames are currently involved in resequencing studies with several nucleoside transporters, cytidine deaminase, dCMP deaminase, deoxycytidine kinase, and deoxycytodilate kinase. Functional polymorphisms in these genes will be correlated with the gemcitabine phenotype studies.

Add 4 14.2 Gemcitabine phenotypic studies

After gemcitabine is transported into cells, the parent drug is sequentially phosphorylated to the triphosphate (dFdCTP). dFdCTP is incorporated into DNA and this mechanism is believed to be associated with drug response. Cellular measurements of dFdCTP reflect the aggregate consequences of cellular uptake, anabolism and catabolism. The hypothesis of this phenotypic study is that intracellular triphosphates in the mononuclear cells will reflect variation in the transport, anabolism and catabolism of gemcitabine as a consequence of genetic variation in the genes and protein products associated with those processes. This pharmacologic or intervening phenotype will be used for correlation with genotypes determined in this study. Gemcitabine concentrations will be measured in the plasma and dFdCTP concentrations will be measured in the mononuclear cells. The red cells will be evaluated for the presence of dFdCTP in measurable concentrations. With a 30 minute infusion of gemcitabine, dFdCTP concentrations reach peak at approximately 90 minutes (M. Tempero et al, J. Clin. Onc., 21:3402-08, 2003).

Add 4

Add 4,5 14.3 Blood Sample Preparation, Storage and Shipping :

14.31 Blood Collection Time Points

Add 2,4,5 14.311 Pharmacogenetics Sample Collection (One sample collected at single time point)

Add 5 1) Prior to treatment

Add 4,5 14.312 Gemcitabine phenotype studies (Two samples collected at 3 time points):

1) Pre-infusion

2) 30 minutes (end of infusion)

3) 90 minutes after the start of infusion or 60 minutes after completion of infusion

Add 5 14.32 Blood Volume Collection/Processing – NOTE: See kit instructions for preparation.

Add 2,4,5 14.321 Collect one 10 ml of whole blood in an EDTA tube (lavender top) prior to treatment and immediately refrigerate sample.

Add 5 14.322 Collect whole blood in 2-10 ml tubes (one heparinized tube - green top, one CPT tube - tiger top) each containing 5 micromole/L tetrahydrouridine (THU; see kit instructions) at the following time points:

- pre-infusion
- 30 minutes (end of infusion)
- 90 minutes after start of infusion (60 minutes after completion of infusion).

Process all samples according to kit instructions. Plasma, buffy coat, and red cells will be isolated from the heparin samples and buffy coat will be isolated from the CPT samples. Immediately freeze specimens at -20°C

or colder. Once all samples have been processed, ship all specimens according to shipping instructions (see 14.33).

Add 5 14.33 Shipping

Add 5,6 14.331 Kits are required for this study. Participating institutions may obtain kits by faxing the Supply Order Form (found in the Forms Packet).

14.3311 Kits will be sent via FedEx® Ground at no additional cost to the participating institutions. **Allow at least two weeks to receive the kits.**

14.3312 Kits will not be sent via rush delivery service unless the participating institution provides their own FedEx® account number or alternate billing number for express service. **NCCTG will <u>not</u> cover the cost for rush delivery of kits.**

14.332 Ship specimens and NCCTG Blood Specimen Submission Form to Mayo Central Laboratory for Clinical Trials (MCLCT) as follows:

Add 5 14.3321 Ship EDTA tube with a solidly frozen cold pack (see kit instructions for proper packing of blood and cold pack to avoid freezing of specimen.)

Add 5 14.3322 Ship frozen samples (i.e., three tubes each of plasma, buffy coat, and red cells isolated from the three heparin tubes, and three tubes each of buffy coat isolated from the three CPT tubes) on dry ice.

Add 5 14.333 All samples should be collected **Monday-Thursday ONLY. Do not send samples on weekends or holidays.**

Add 5 14.334 Make sure the specimen tubes are correctly labeled with patient initials, NCCTG patient ID number, protocol number, and time and date drawn.

Add 5 14.335 ALL sections of the form/specimen collection labels must be completed.

Add 5 14.336 **<u>NOTE</u>:** A small, but sufficient, supply of the specimen collection kits should be ordered prior to patient entry.

Add 5 14.337 **<u>NOTE</u>:** The kit contains instructions for collecting and processing specimens for shipping.

Add 5 14.338 Use kit mailing labels for shipment to MCLCT.

Add 5,6 14.339 MCLCT will receive the samples and forward specimens within two hours of receipt to the NCCTG Research Base Biospecimens Accessioning and Processing (BAP) Shared Resource, Stabile 13-10A, attention BAP Supervisor.

Add 5 14.3391 BAP will record receipt of pretreatment EDTA sample. BAP will isolate the DNA, immediately aliquot 10 ul (before quantification) for UGT1A1 genotyping in the Matthew Ames' Laboratory and store the remaining DNA indefinitely. BAP

should contact Stephanie or Mary (507-284-4303) in Dr. Ames' laboratory for immediate pickup of the 10 ul aliquot and the frozen specimens. UGT1A1 results will be reported in three business days from sample receipt. UGT1A1 genotyping results will be faxed from Dr. Ames' laboratory to the NCCTG member site, NCCTG randomization office, and the principal investigator. As additional relevant functional polymorphisms are identified, future analyses will be conducted depending on the patient consent permission.

Add 5 14.3392 BAP will record receipt of all specimens for the gemcitabine phenotype studies. BAP will contact Stephanie or Mary (507-284-4303) in Dr. Ames' laboratory for immediate pickup of the frozen specimens (i.e., three tubes each of plasma, buffy coat, and red cells isolated from the three heparin tubes, and three tubes of buffy coat isolated from the three CPT tubes).

14.34 Banking of tumor tissue for future research of tissue correlates of response and/or toxicity: Submission of tissue samples for research testing is mandatory.

Add 5

14.341 The following materials are required for tissue samples submitted for this protocol:

Add 2,4,5

- NCCTG Tissue Specimen Submission Form
- A copy of the operative and pathology reports.
- At least one (three, if possible) paraffin-embedded blocks with representative tumor. The formalin fixed tissue sample is preferred; however, if an institution is unable to release tissue blocks, they must be willing to submit 6 unstained charged slides cut at 5 microns.

Add 2,4,5

14.342 The blocks will be available upon specific request to accommodate individual patient management. The institutional pathologist must be notified that the block may be depleted. At the completion of the study, remaining blocks will be retained in the NCCTG Operations Office.

Add 2,4,5,6 14.343 Additional stains will be performed on all tissue for eligible patients. These are investigational only, and results will be used in a separate analysis. These stains include: CK7 and CK20 profile. Certain patterns of staining are characteristic of primary sites, but not specific enough for diagnosis (52). We will determine if particular staining combinations are associated with response rate in an exploratory
Add 6 manner (there are too few patients to perform a planned subset analysis). Samples will be analyzed in the labs of Marie Christine Aubry, M.D. and Timothy F. Drevyanko, M.D.

Add 6 14.344 92 gene RT-PCR cancer classification assay. Blinded, de-identified, and de-linked cancer samples in the form of three 5 micron unstained tissue sections plus an adjacent H&E stained section will be received by AviaraDx from the Mayo Clinic. The H&E stained section will be examined by either a designated pathologist at Mayo Clinic or AviaraDx's medical director (Bernie Chang, M.D.) to confirm the presence of cancer as well as outline the cancerous area with a "sharpie pen" to facilitate subsequent macrodissection of the tumor of interest.

The 92-gene RT-PCR tumor panel includes 87 target genes and 5 reference genes (see attached publication for gene list: Ma et al., Arch. Path. Lab. Med.). Each sample is analyzed for these 92 genes by TaqMan real-time PCR on the ABI 7900 on a 384-well plate. For each

gene, PCR is carried out in a single well. The raw C_T values exported from ABI 7900 to a text file are used for further analysis.

Add 2,4,5,6

14.345 Blocks or slides should be placed in individual plastic bags and each bag labeled with the cooperative group membership name, study patient number (local ID and NCCTG enrollment number), patient's initials, protocol number, surgical accession number, and source (e.g., primary).

Add 2,4,5,6

14.346 The material will be stored for future research depending on the patient consent permission (see Section 6.3) in order to be suitable for immunohistochemistry (IHC) and to perform DNA extraction. When a protocol is developed, it will be presented for IRB review and approval. The blocks may also be used to construct tissue microarrays (TMAs) for future studies. TMAs will be analyzed to assess predictive biomarkers, changes in expression pattern with therapy, and correlation with response and/or adverse events. The donor block remains intact except for 6 small (0.6mm) holes where the cores were taken. This process has minimal impact on the utility of the block for future clinical diagnostic needs.

Add 1,2,4,5,6

14.347 Submit the required materials within 30 days following registration to the following address.

NCCTG Operations Office
ATTN: NCCTG PC Office
RO_FF_03_24-CC/NW Clinic
200 First Street SW
Rochester, MN 55905

15.0 Drug Information

15.1 Gemcitabine-HCL (GEMZAR)

15.11 Preparation and storage: GEMZAR is supplied as a lyophilized powder in 200 mg and 1000 mg vials. Store intact vials at room temperature, do not refrigerate. To reconstitute, add 5 mL of 0.9% Sodium Chloride Injection to the 200 mg vial or 25 mL of 0.9% Sodium Chloride Injection to the 1 g vial. Shake to dissolve. These dilutions each yield a gemcitabine concentration of 38 mg/mL. The total volume upon reconstitution will be 5.26 mL or 26.3 mL, respectively. The reconstituted solution contains no preservatives and should be used within 24 hours. IV solutions in 250 mL NS are stable for 24 hours. IV infusion in 250 mL NS over 30 minutes. (The maximum final concentration should not exceed 40 mg/mL - may dilute up to 400 mL NS if needed). Do not use other diluents. The pH of the final solution is around 3. There may be some local irritation due to the low pH (pain at injection site). Pediatric products should be restricted to a final volume less than 200mL/m2 to avoid volume overload. GEMZAR is not a vesicant.

15.12 Known potential toxicities:

15.121 Hematologic: Myelosuppression (neutropenia, anemia, thrombocytopenia).

15.122 Dermatologic: Alopecia, transient mild erythematous pruritic rash, desquamation, Stevens Johnson Syndrome.

15.123 Gastrointestinal: Nausea, vomiting, anorexia, diarrhea.

15.124 CNS: Somnolence, agitation, insomnia, dizziness, paresthesia, confusion, convulsion, coma.

Add 2

15.125 Renal: Renal dysfunction (increased BUN and creatinine values, proteinuria, hematuria, anuria), kidney damage, hemolytic uremic syndrome (HUS).

Add 2

15.126 Other: Headache, shortness of breath, pneumonitis, mild chills and fever, arthralgias, tachycardia, flu-like symptoms, asthenia, malaise, and fatigue, weakness with or without myalgia, cardiac insufficiency, irregular heartbeat, elevated bilirubin, capillary leak syndrome, change in blood chemistry.

Add 3

15.127 Lilly recommends a two-week delay between the conclusion of radiation and the start of gemcitabine, provided the acute effects of radiation treatment have resolved.

15.13 Nursing guidelines:

15.131 Monitor CBC, differential, PLTs prior to each dose. Myelosuppression is the principal dose-limiting factor. Modification may be considered by physician when bone marrow suppression is suspected.

15.132 Evaluate hepatic and renal function prior to initiation of therapy and periodically thereafter. Closely observe those patients with a history of preexisting mild renal impairment or hepatic insufficiency. Encourage hydration.

15.133 GEMZAR clearance is affected by age and gender. Grade 3/4 thrombocytopenia has been more common in elderly women.

15.134 Antiemetics may be required for probable mild to moderate nausea and vomiting.

15.135 Instruct patient in management of possible mild diarrhea and stomatitis.

15.136 GEMZAR may cause fever in the absence of clinical infection. Fever can be accompanied by other flu-like symptoms. Instruct patient to report fever or flu-like symptoms to healthcare team. Treat symptoms as they occur.

15.137 Macular or finely granular maculopapular eruptions were experienced by 30% of patients tested. Instruct patients to report any skin changes.

Add 2

15.138 Instruct patient to report any cough, shortness of breath, or chest pain immediately.

Add 2

15.139a Burning may occur at the injection site. May apply heat during infusion to minimize pain.

Add 2

15.139b The diagnosis of HUS should be considered if the patient develops anemia with evidence of microangiopathic hemolysis as indicated by elevation of bilirubin or LDH, reticulocytosis, severe thrombocytopenia, and/or evidence of renal failure (elevation of serum creatinine of BUN). Gemzar therapy should be discontinued immediately. Renal failure may not be reversible even with discontinuation of therapy and dialysis may be required.

Add 3

15.14 Drug procurement: Eli Lilly will supply investigational drug to the NCCTG Coordinating Center Pharmacy. Each institution will order the drug from the NCCTG Coordinating Center Pharmacy by submitting an NCCTG Clinical Drug Request Form. The form can be found at https://ncctg.mayo.edu/ncctg/forms/NonProtocolSpecificForms/ and should be submitted to:

Medical Oncology Pharmacist
Mayo Clinic
Gonda 10-178
Rochester, MN 55905
Fax (507) 284-3464

15.2 Irinotecan/CPT-11 (Camptosar®) (Commercial supply)

15.21 Formulation and storage: Irinotecan hydrochloride trihydrate (CPT-11, Camptosar®) is supplied in amber single-dose vials and appears as a pale yellow transparent solution. Two vial sizes are available: 2 mL vials containing 40 mg of drug and 5 mL vials containing 100 mg of drug. Store at controlled room temperature 15-30°C (59-86°F). Vials should be kept in the carton, protected from light, and kept in a secured area, accessible only to authorized personnel, until the time of use.

15.22 Preparation: The calculated dose is diluted in 500 mL of D_5W.

15.23 Compatibility: No other drug should be mixed with irinotecan.

15.24 Stability: Irinotecan, in the vials, is stable for at least 3 years at room temperature. After reconstitution with D_5W, in glass bottles or plastic bags, the diluted solution is stable for at least 24 hours at room temperature and in ambient lighting and 48 hours at refrigerated temperatures.

15.25 Administration: Following reconstitution, irinotecan should be administered as an intravenous infusion over 90 minutes.

15.26 Known potential toxicities:

- Hematologic - myelosuppression (neutropenia, leukopenia including lymphocytopenia and anemia). Virtually all phase I and II studies of irinotecan have reported neutropenia as a dose-limiting toxicity. Thrombocytopenia is uncommon.

- Hypersensitivity reactions - hypotension and/or bronchospasm or generalized rash/erythema, flushing, rash with or without pruritus, chest tightness, back pain, dyspnea, drug fever, and chills.
- Dermatologic - Alopecia. A generalized rash with or without pruritis may occur. Irinotecan may cause local irritation at the infusion site. Extravasation necrosis of the skin has not been reported in US studies.

- Gastrointestinal - nausea, vomiting, diarrhea. Irinotecan can induce both early and late forms of diarrhea that appear to be mediated by different mechanisms. Both forms of diarrhea may be severe. Late diarrhea has been dose limiting in some phase I and II studies. Early diarrhea (occurring during or within 8 hours of administration) may be preceded by complaints of diaphoresis, abdominal cramping, and lacrimation. This syndrome is thought to be cholinergically mediated and may be ameliorated by atropine. Diarrhea or abdominal cramping that occurs during or within one hour after receiving irinotecan should be treated with 0.25 to 1 mg of atropine IV (unless there is a relative or absolute contraindication to its use such as, but not limited to, closed-angle glaucoma, urinary retention/obstructive uropathy, or previous untoward reactions to the administration of atropine). Late diarrhea (occurring more than 8 hours after administration of CPT-11) can be prolonged, may lead to dehydration and electrolyte imbalance, and can be life-threatening. Late diarrhea should be treated promptly with loperamide (suggest: loperamide 4 mg at first onset of diarrhea, then 2 mg q2h around the clock until diarrhea free for at least 12 hours. If diarrhea is not controlled after 3 days, loperamide should be stopped, and hydration and hospitalization should be considered). Patients with severe diarrhea should be carefully monitored and given fluid and electrolyte replacement if they become dehydrated. Infrequent occurrences of mucositis or colitis, sometimes with gastrointestinal bleeding, have been reported.
- Cardiovascular - vasodilation (flushing).
- Hepatic - Occasional liver enzyme abnormalities.
- Respiratory - Bronchospasms, non-productive cough, transient infiltrates, and dyspnea.
- Neurologic - Insomnia and dizziness. (Dizziness may sometimes have represented symptomatic evidence of orthostatic hypotension in patients with dehydration).
- Renal - Occasional abnormalities of the serum creatinine have been reported.
- Body as a whole - abdominal pain and asthenia.

Add 7 Per review of Irinotecan (CPT-11) package insert, the following potential risks have been added: constipation, anorexia, excessive saliva, and pancreatitis.

15.27 Drug procurement: Commercially available.

15.28 Nursing implications:

15.281 If possible, check for any history of hypersensitivity reaction to any previous drug formulated with polysorbate 80.

15.282 Cholinergic symptoms of lacrimation, nasal congestion, diaphoresis, flushing, ABD cramping, and diarrhea can occur at the beginning, during, or immediately after the CPT-11 infusion. It is suggested that the patient remain in the treatment area for a minimum of one hour following the completion of the **very first** CPT-11 infusion. If diarrhea occurs within one hour of infusion, refer to Section 9.2 for management.

15.283 Patient education is extremely important. Impress on the patient the importance of compliance with treatment of diarrhea management (see Section 9.1 and Appendix III). Stress the need for prompt recognition and early intervention. Motivate the patient to report any complications immediately. The cholera-like syndrome can be unresponsive to conventional antidiarrheals and can result in severe dehydration.

15.284 Ondansetron and diphenhydramine should provide good relief from the nausea/vomiting/cramping. Avoid prochlorperazine on the day of treatment due to its association with akathisia (motor restlessness). Prochlorperazine may be taken between treatments.

15.285 Advise avoidance of excess caffeine, a GI stimulant. Avoid magnesium based antacids such as Mylanta, Maalox, Rolaids, MOM, Mag-Ox 400, and Tylenol with antacid.

15.286 The pulmonary toxicity seen is usually manifested by dyspnea beginning 42-175 days after treatment and occurs at a cumulative dose ranging from 400-1000 mg/m (median 750). Instruct patient to report any cough or SOB.

15.287 Patients are at risk for developing eosinophilia and will improve on steroid therapy.

15.288 Hepatic enzyme elevations have been transient and did not require intervention.

15.289a Monitor CBC closely. Leukopenia occurs primarily as neutropenia but can be severe and dose-limiting. The simultaneous occurrence of grade 4 diarrhea and grade 4 neutropenia is rare but may render the patient more susceptible to polymicrobial sepsis and potentially death.

15.289b Advise patients of probable hair loss.

16.0 Statistical Considerations and Methodology

Add 1,4

16.1 Overview: As of Addendum 4, this is the new study design to use for this study. The original study design is listed starting in section 16.6. We developed a new study design because (1) the accrual rate was found to be much lower than expected (1/mo vs. 3-4/mo) and (2) due to the level of toxicity observed for cohort I (regardless of genotype), a recommendation was made to reduce the gemcitabine dose and to reduce the cycle from 6 weeks to 4 weeks by dropping the last day of treatment (d22). Due to these major changes to the treatment schedule, we redesigned the study by reducing the overall sample size and to assess (for efficacy), only Cohort II patients. Cohort II consists of all patients accrued on or after the Addendum 4 effective date. This new design is set-up as a one-stage phase II study with an interim analysis. This study will assess the toxicity and confirmed response rate associated with the treatment regimen of gemcitabine and irinotecan for patients with unknown primary carcinoma.

Add 4

16.11 Primary Endpoint: The primary endpoint is confirmed response rate. If measurable disease is present, a confirmed tumor response is defined to be either a CR or PR noted as the objective status on 2 consecutive evaluations at least 4 weeks apart (see Section 11.0). All registered patients meeting the eligibility criteria that have signed a consent form and have begun treatment will be evaluable for response.

Add 1,4,5

16.12 Sample Size: The study design to be utilized is fully described in Section 16.2. A minimum of 13 to a maximum of 25 evaluable patients will be accrued unless undue adverse events are encountered. We anticipate accruing an additional 3 patients to account for ineligibility, cancellation, major treatment violation, or other reasons (see 16.14). Therefore, the maximum possible accrual for Cohort II is 28 patients. Patients accrued to Cohort I on or after the Addendum 4 effective date will be added to Cohort II as noted in Section 16.1; therefore total sample size will be 42 patients (i.e., previously accrued Cohort I patients[=14 patients] plus Cohort II patients [=28 patients]).

Add 4

16.13 Accrual Time and Study Duration: The anticipated accrual rate is approximately 1 patient per month based on the accrual to this study for the Cohort I analysis. Therefore, the accrual period for this phase II study is expected to be about 28 months. The final analysis can begin approximately 32 months after the trial begins (i.e., as soon as the last patient has been observed for at least 4 cycles of 4 weeks each).

Add 4

16.14 Toxicity Stopping Rule: We'll assess the first 6 evaluable patients in each genotype group at the new Addendum 4 dose level and treatment schedule. We expect about 50% of patients to carry the UGT1A1*28 polymorphism, and the other 50% to be negative for it. With this in mind, we expect to only have to accrue about 15 patients during this phase to get 6 patients in each group (i.e., 6 patients with the UGT1A1*28 polymorphism, and 6 without it). If the trial does meet the criteria for efficacy, we will determine the frequency and nature of the cycle 1 adverse events. If the overall

Add 6

frequency of cycle 1 grade 4+ non-hematologic adverse events (at least possibly related to treatment) is $\geq$ 33% across all patients, consideration will be given to stopping the trial. If however, the frequency of grade 4+ non-hematologic adverse events is acceptable (< 33%), we will go on to determine the incidence of the following cycle 1 hematologic adverse events (regardless of attribution): Grade 4+ neutropenia, grade 3+ febrile neutropenia and grade 3+ infection in the presence of grade 3 or 4 neutrophils. The following scenarios apply for assessment of these hematologic adverse events:

1) If ≥33% of UGT1A1 6/7 or 7/7 patients experience at least one of the above cycle 1 hematologic adverse events while < 33 % of UGT1A1 6/6 patients experience these cycle 1 adverse events, an addendum will be written to establish genotype-based dosing with a reduced dose for patients who carry the UGT1A1*polymorphism.
2) If in the overall group (all patients) the incidence of these cycle 1 hematologic adverse events is ≥33% and there does not exist a clear relationship with UGT1A1 genotype, consideration will be given to closing the trial at that time.
3) If in the overall group (all patients) the incidence of these cycle 1 hematologic adverse events <33%, and there does not exist a clear relationship between these adverse events and UGT1A1 genotype, the trial will continue at the same dose, regardless of genotype.

Add 5 As the trial proceeds beyond the initial assessment of adverse events described above, we will continue to use the following adverse event stopping rules in all patients. If either (1) or (2) occurs below, we will suspend the trial and do a full review of the data.

Add 6

Add 5
1) If the overall frequency of grade 4+ non-hematologic adverse events (at least possibly related to treatment) is $\geq$ 33% across all patients

Add 5
2) Hematologic adverse events (regardless of attribution) is ≥50% in all patients: Grade 4+ neutropenia, grade 3+ febrile neutropenia and grade 3+ infection in the presence of grade 3 or 4 neutrophils.

Add 5

Additionally, all grade 5 events will be reviewed on a case-by-case basis and the trial will be suspended if at any time 2 or more grade 5 events occur that are at least possibly related to the study medication at the appropriate Cohort II dose levels.

16.2 Statistical Design:

Add 4
16.21 Overview: This study is designed to look at confirmed response rate in patients with unknown primary carcinoma. Based upon the history of this disease (50) we would expect at most 20% of patients to have a confirmed response. For an agent to provide significant clinical improvement from standard therapy, the confirmed response rate should be at least 40%.

Add 4
16.22 Definition of a Success: A success is defined as a patient who has a confirmed response as defined in Sections 11.0 and 16.11.

Add 1,4
16.23 Decision Rule: The largest success proportion where the proposed treatment regimen would be considered ineffective in this population is 20%, and the smallest success proportion that would warrant subsequent studies with the proposed regimen in this patient population is 40%. The following Three-Outcome Phase II study design (51) with an interim analysis uses a maximum of 25 patients to test the null hypothesis that the true success proportion in a given patient population is at most 20%. An interim analysis will be performed at the time the 13th patient becomes evaluable for the Addendum 4 dose level. If at least 3 successes are observed in these initial 13 evaluable patients (regardless of genotype for those patients receiving the Addendum 4 dose level), we will continue enrollment to a maximum of 25 evaluable patients (regardless of genotype or dose level after the dose was changed for Addendum 4). Otherwise, we will discontinue enrollment and conclude that the regimen is not sufficiently active in this patient population.

Add 1,4
16.231 "Not promising": This regimen will be classified as not promising with respect to the success rate in this patient population if at most 6 successes are observed in a total of 25 evaluable patients.

Add 1,4
16.232 "Inconclusive": The results of this study will be classified as inconclusive with respect to this regimen demonstrating an improved success rate if 7 successes are observed in 25 evaluable patients. In this case, toxicity, quality of life, and duration of response observed in this

study will be used in addition to confirmed response rate to make the final determination as to whether or not this treatment is considered promising and worthy of further study in this patient population.

Add 4 16.233 "Promising": This regimen will be classified as promising with respect to increasing the success rate in this patient population if at least 8 successes are observed in 25 evaluable patients. Subsequent larger confirmatory studies may be recommended.

Add 4 16.24 Power and Significance Level: Assuming that the number of successes is binomially distributed, the significance level is 0.10 and the probability of declaring that this regimen warrants further studies (i.e., statistical power) under various success proportions can be tabulated as a function of the true success proportion as shown in the following table:

Add 1,4

If the true success proportion is	0.20	0.25	0.30	0.35	0.40
then the probability of declaring that the regimen warrants further studies is	0.10	0.26	0.47	0.67	0.83
and the probability of stopping at the interim analysis is	0.50	0.33	0.20	0.11	0.06
and the probability of determining that the study is inconclusive is	0.09	0.14	0.14	0.11	0.07

Add 1,4 In other words, this design has approximately 83% power to detect a true response rate of at least 40%, and there is only a 7% chance of having an inconclusive result given that the true response rate is 40%.

Add 4 16.25 Other Considerations: Toxicity, quality/duration of response, and patterns of treatment failure observed in this study, as well as scientific discoveries or changes in standard care, will be taken into account in any decision to terminate the study.

Add 4 16.3 Analysis Plan:

Add 4 16.31 Primary Endpoint: The proportion of successes will be estimated by the number of successes divided by the total number of evaluable patients. All evaluable patients will be followed until death or a maximum of 2 years from the time of registration to this trial.

16.32 Definitions and Analyses of Secondary Endpoints:

Add 4 16.321 Overall survival time is defined as the time from registration to death due to any cause. The distribution of survival time will be estimated using the method of Kaplan-Meier (50). Overall survival will be calculated for all evaluable patients combined and by group (ie. for patients with or without the UGT1A1*28 polymorphism).

Add 4 16.322 Time to disease progression is defined as the time from registration to documentation of disease progression. If a patient dies without a documentation of disease progression, the patient will be considered to

have had tumor progression at the time of their death unless there is sufficient documented evidence to conclude no progression occurred prior to death. If the patient is declared to be a major treatment violation, the patient will be censored on the date the treatment violation was declared to have occurred. In the case of a patient starting treatment and then never returning for any evaluations, the patient will be censored for progression on day 1 post-registration**.** The distribution of time to progression will be estimated using the method of Kaplan-Meier (50). Time to disease progression will be calculated for all evaluable patients combined and by group (ie. for patients with or without the UGT1A1*28 polymorphism).

Add 4

16.33 Toxicity:
As per NCI CTCAE v3.0, the term toxicity is defined as adverse events that are classified as either possibly, probably, or definitely related to study treatment. The maximum grade for each type of adverse event will be recorded for each patient, and frequency tables will be reviewed to determine adverse event patterns. In addition, we will review all adverse event data that is graded as 3, 4, or 5 and classified as either "unrelated or unlikely to be related" to study treatment in the event of an actual relationship developing. Toxicity will be calculated for all evaluable patients combined and by group (ie. for patients with or without the UGT1A1*28 polymorphism).

Add 4

16.34 Over Accrual:
If more than the target number of patients are accrued, the additional patients will not be used to evaluate the stopping rule or used in any decision-making processes; however, they will be included in final point estimates and confidence intervals.

Add 4

16.35 Routine Monitoring:
Efficacy, toxicity, and administrative information for this trial will be reviewed by the study team twice per year in conjunction with production of the semiannual NCCTG Group Meeting reports. They will monitor the trial for evidence of severe adverse events and feasibility problems.

Add 4

16.4 Translational Component: A blood sample will be collected ≤14 days prior to registration to assess whether variation in multiple different genes whose protein products are involved in the uptake, metabolism, and distribution of Gemzar and CPT-11 affect clinical outcomes (i.e., response, toxicity, survival, time to progression, etc.). All analyses with respect to the translational component of this study are intended to be hypothesis-generating and descriptive in manner. The correlations between gene type (obtained at baseline) and response (and toxicity) will be explored via frequency tables (i.e., Chi-square analyses). The correlation of gene type with other patient outcomes (i.e., survival, time to progression, etc.) will be explored via Cox proportional hazards regression (52). The distributions of survival, and time to progression defined by the different genes will be estimated via Kaplan-Meier methodology (50).

The translational component of this study also proposes to stain all specimens for CK7/20 in an attempt to ascertain which patterns may be predictive of response to this regimen. This will be an exploratory analysis, as the numbers in this phase II study are too small to make any firm conclusions. We will also request the tissue blocks be retained here so

that we may pursue additional translational studies in the future in conjunction with our pathology colleagues.

Add 6 In addition, we plan to assess the relationship between the site of origin (by gene expression profiling) and response/time-to-progression.

Add 4 16.5 Inclusion of Women and Minorities

Add 4 16.51 This study will be available to all eligible patients, regardless of race, gender, or ethnic origin.

Add 4 16.52 There is no information currently available regarding differential effects of this regimen in subsets defined by race, gender, or ethnicity, and there is no reason to expect such differences to exist. Therefore, although the planned analysis will, as always, look for differences in treatment effect based on racial and gender groupings, the sample size is not increased in order to provide additional power for subset analyses.

Add 4 16.53 Based on prior studies involving similar disease sites, we expect about 5% of patients will be classified as minorities by race and about 33% of patients will be women. Estimates of racial by gender subsets are shown in the following table (assuming the maximum sample size of 28 (including over accrual) is reached):

Ethnic Category	Sex/Gender			
	Females	Males	Unknown	Total
Hispanic or Latino	0	1	0	1
Not Hispanic or Latino	9	18	0	27
Unknown	0	0	0	0
**Ethnic Category: Total of all subjects**	9	19	0	**28**
**Racial Category**				
American Indian or Alaskan Native	0	0	0	0
Asian	0	0	0	0
Black or African American	0	1	0	1
Native Hawaiian or other Pacific Islander	0	0	0	0
White	9	18	0	27
More than one race	0	0	0	0
Unknown	0	0	0	0
**Racial Category: Total of all subjects**	9	19	0	**28**

Add 4

Ethnic Categories: **Hispanic or Latino –** a person of Cuban, Mexican, Puerto Rican, South or Central American, or other Spanish culture or origin, regardless of race. The term "Spanish origin" can also be used in addition to "Hispanic or Latino."

Not Hispanic or Latino

Racial Categories: **American Indian or Alaskan Native –** a person having origins in any of the original peoples of North, Central, or South America, and who maintains tribal affiliations or community attachment.

Asian – a person having origins in any of the original peoples of the Far East, Southeast Asia, or the Indian subcontinent including, for example, Cambodia, China, India, Japan, Korea, Malaysia, Pakistan, the Philippine Islands, Thailand, and Vietnam. (Note: Individuals from the Philippine Islands have been recorded as Pacific Islanders in previous data collection strategies.)

Black or African American – a person having origins in any of the black racial groups of Africa. Terms such as "Haitian" or "Negro" can be used in addition to "Black or African American."

Native Hawaiian or other Pacific Islander – a person having origins in any of the original peoples of Hawaii, Guam, Samoa, or other Pacific Islands.

White – a person having origins in any of the original peoples of Europe, the Middle East, or North Africa.

16.6 Original Statistical Design (Prior to Addendum 4)

Add 1,4

Design Overview: This is a one-stage phase II study with an interim analysis and a lead-in phase to assess tolerability. This study will assess the tolerability and confirmed response rate associated with the treatment regimen of gemcitabine and irinotecan for patients with unknown primary carcinoma.

16.61 Primary Endpoint: The primary endpoint is confirmed response rate. If measurable disease is present, a confirmed tumor response is defined to be either a CR or PR noted as the objective status on 2 consecutive evaluations at least 6 weeks apart (see Section 11.0). All registered patients meeting the eligibility criteria that have signed a consent form and have begun treatment will be evaluable for response.

Add 1,4

16.62 Sample Size: The study design to be utilized is fully described in Section 16.62. A minimum of 17 to a maximum of 49 evaluable patients will be accrued unless undue adverse events are encountered. We anticipate accruing an additional 5 patients to account for ineligibility, cancellation, major treatment violation, or other reasons. Therefore, the maximum possible accrual is 54 patients.

Add 4

16.63 Accrual Time and Study Duration: The anticipated accrual rate is approximately 3-4 patients per month. Therefore, the accrual period for this phase II study is expected to be about 18 months. The final analysis can begin approximately 24 months after the trial begins (i.e., as soon as the last patient has been observed for at least 4 cycles).

Add 1,2,4

16.64 Tolerability Assessment (Cohort I phase): We expect about 50% of patients to carry the UGT1A1*28 polymorphism, and the other 50% to be negative for it. With this in mind, we expect to only have to accrue about 15 patients during the Cohort I phase to get 6 patients in each group (i.e., 6 patients in the UGT1A1*28 polymorphism group, and 6 patients in the group without the 28 polymorphism). If more than 15 patients are needed during Cohort I, an addendum will be developed to revise the projected accrual for the Cohort I phase. We anticipate accruing an additional 2 patients to account for ineligibility, cancellation, major treatment violation, or other reasons during Cohort I. Therefore maximum accrual during Cohort I is expected to be 17 patients. All patients enrolled during the Cohort I phase will be treated at dose level 0 (see Section 7.2). After the first 6 evaluable patients have been enrolled into each group (i.e., 6 patients with the UGT1A1*28 polymorphism, and 6 without it), accrual will be suspended, and adverse events will be evaluated before any additional patients will be entered. Only the first 6 evaluable patients entered into each group (i.e., with or without the *28 polymorphism) will be used to determine the appropriate Cohort II dose levels, no matter how many patients end up being enrolled during Cohort I.

Add 1,4

- If the Cohort I starting dose level gets adjusted for both groups (i.e., group 1: patients with UGT1A1*28 polymorphism, group 2: patients without the *28 polymorphism) for the Cohort II phase, a maximum of 37 additional patients (regardless of whether they have the UGT1A1*28 polymorphism or not) will be treated (per study design) at the new dose levels determined during the Cohort I phase (see Section 7.4). Only the patients treated at the appropriate Cohort II dose levels will be included in the decision rule described below in Section 16.23.

Add 1,4

- If the Cohort I starting dose level is adjusted for one group only (i.e., either group 1 or 2), a maximum of 31 additional patients (regardless of whether they have the UGT1A1*28 polymorphism or not) will be treated (per study design) at the dose levels determined during the Cohort I phase (see Section 7.4). Only the patients treated at the appropriate Cohort II dose levels will be included in the decision rule described below in Section 16.23.

Add 1,4

- If the Cohort I starting dose level is not adjusted for either group, a maximum of 25 additional patients will continue to be treated (per study design) at dose level 0 (see Section 7.2), and all patients will be included in the decision rule described below in Section 16.23.

Add 4

16.7 Statistical Design:

Add 4

16.71 Overview: This study is designed to look at confirmed response rate in patients with unknown primary carcinoma. Based upon the history of this disease (50) we would expect at most 20% of patients to have a confirmed response. For an agent to provide significant clinical improvement from standard therapy, the confirmed response rate should be at least 40%.

Add 4

16.72 Definition of a Success: A success is defined as a patient who has a confirmed response as defined in Sections 11.0 and 16.11.

Add 4

16.73 Decision Rule: The largest success proportion where the proposed treatment regimen would be considered ineffective in this population is 20%, and the smallest success proportion that would warrant subsequent studies with the proposed regimen in this patient population is 40%. The following Three-Outcome Phase II study design (51) with an interim analysis u[illegible] Addenda 1,4 of 37 patients to test the null hypothesis that the true success prop[illegible] patient population is at most 20%. An interim analysis will be performed at the time the 17th patient becomes evaluable for the appropriate Cohort II dose levels. If at least 4 successes are observed in these initial 17 evaluable patients, we will continue enrollment to a maximum of 37 evaluable patients treated at the appropriate Cohort II dose levels. Otherwise, we will discontinue enrollment and conclude that the regimen is not sufficiently active in this patient population.

Add 1,4

16.731 "Not promising": This regimen will be classified as not promising with respect to the success rate in this patient population if at most 10 successes are observed in a total of 37 evaluable patients.

16.732 "Inconclusive": The results of this study will be classified as inconclusive with respect to this regimen demonstrating an improved success rate if 11 successes are observed in 37 evaluable patients. In this case, toxicity, quality of life, and duration of response observed in this study will be used in addition to confirmed response rate to make the final determination as to whether or not this treatment is considered promising and worthy of further study in this patient population.

16.733 "Promising": This regimen will be classified as promising with respect to increasing the success rate in this patient population if at least 12 successes are observed in 37 evaluable patients. Subsequent larger confirmatory studies may be recommended.

16.74 Power and Significance Level: Assuming that the number of successes is binomially distributed, the significance level is 0.05 and the probability of declaring that this regimen warrants further studies (i.e., statistical power) under various success proportions can be tabulated as a function of the true success proportion as shown in the following table:

Add 1

If the true success proportion is	0.20	0.25	0.30	0.35	0.40
then the probability of declaring that the regimen warrants further studies is	0.05	0.18	0.42	.67	0.85
and the probability of stopping at the interim analysis is	0.55	0.35	0.20	0.10	0.05
and the probability of determining that the study is inconclusive is	0.05	0.10	0.12	0.10	0.05

Add 1 In other words, this design has approximately 85% power to detect a true response rate of at least 40%, and there is only a 5% chance of having an inconclusive result given that the true response rate is 40%.

Add 2

17.0 Pathology Considerations

Add 2 17.1 Pathology review for those institutions **who cannot confirm** histology by H&E and **cannot perform** the stains as outlined in Section 3.12.

17.11 Required materials

- Paraffin blocks containing tumor tissue from the most recent tumor tissue biopsy. If the institution is unable to provide a block, submit 15 regular charged slides (5 microns).
- Tumor tissue diagnostic H&E stained slide
- Tumor tissue pathology report
- Pathology Reporting Form

Blocks/slides should be placed in individual plastic bags and each bag labeled with the protocol number, study patient number, patient initials, and surgical accession number.

Add 5,6 17.12 All materials listed above must be sent directly to the following address:

NCCTG Operations Office
ATTN: NCCTG PC Office
RO_FF_03_24-CC/NW Clinic
200 First Street SW
Rochester, MN 55905

Add 7 **In order to assure prompt handling, please call Christine Maszk at (507) 266-8919 to alert of the time/date sent and courier contracted.**

Marie Christine Aubry, M.D., will review the slides for confirmation of disease.

17.13 The NCCTG Pathology Coordinator will take the blocks/slides to NCCTG Tissue Acquisition and Cellular/Molecular Analysis Shared Resource laboratory. Once the blocks/slides are prepared Dr. Aubry will receive them for review. The NCCTG Pathology Coordinator will call the institution notifying them of the results. The NCCTG Pathology Coordinator will fax a copy of the completed

Pathology Reporting Form to the randomizing member. If eligibility was confirmed, the NCCTG Pathology Coordinator will complete a NCCTG Pathology Eligibility Documentation Form and forward it to the Randomization Center. Upon receipt of the NCCTG Pathology Eligibility Documentation Form, the Randomization Center can register the patient.

Add 2 17.2 Pathology review for all other institutions **who can confirm** histology **or perform** the stains as outlined in Section 3.12.

17.21 Required materials to be submitted ≤30 days of registration:

Add 2
- Paraffin block containing tumor tissue from the most recent tumor tissue biopsy. If the institution is unable to provide a block, submit 15 regular charged slides (5 microns).
- Diagnostic slides (if stains were done, slides of those stains need to be submitted)
- Tumor tissue diagnostic H&E stained slide
- Tumor tissue pathology report
- Pathology Reporting Form

Blocks/slides should be placed in individual plastic bags and each bag labeled with the protocol number, study patient number, patient initials, and surgical accession number.

Add 5, 6 17.22 All materials listed above must be sent directly to the following address:

NCCTG Operations Office
ATTN: NCCTG PC Office
RO_FF_03_24-CC/NW Clinic
200 First Street SW
Rochester, MN 55905

Marie-Christine Aubry, M.D., will review the slides for confirmation of disease.

17.23 If pathology review finds the patient ineligible based on the criteria, this patient will be considered a protocol violation but may continue on study. All slides, remaining paraffin block, and forms will be returned to the submitting institution.

	Forms	Active-Monitoring Phase (Compliance with Test Schedule)					Event-Monitoring Phase[1] (Completion of Active-Monitoring Phase)				At Each Occurrence			
		Initial Material		Pathology Review	Follow-up material									
		≤2 weeks prior to registration	≤2 weeks after registration	≤30 days after registration	At each evaluation	At end of treatment	Every 3 months until PROG[1]	At PROG[1]	After PROG q. 3 months[1]	Death	ADR/ AER	New Primary	Grade 4 or 5 Non-AER Reportable Events/ Hospitalization	Late Adverse Event
Add 2	On-Study Form		X											
	OP and Path Reports		X											
	Measurement Form		X		X	X								
Add 2,4	UGT1A1 Submission/Results Form[4]	X[3,4]												
	Baseline Specimen Submission Form-Blood	X[3,4]												
	Cohort II Specimen Submission Form -Blood				X[5]									
Add 2,5	Pathology Reporting Form (see Section 17.0)			X										
	Specimen Submission Form-Tissue (see Section 14.34)			X										
	Interval Lab Form				X[2]									
	Event-Monitoring Form						X	X	X	X		X		X
Add 4	Evaluation/Treatment Form-Cohort I				X	X								
	Evaluation/Treatment Form-Cohort II				X	X								
	Adverse Event Log				X	X								
	End of Active Treatment Form					X								
	ADR/AER (See Section 10.0)										X			
	Secondary AML/MDS Report Form											X		
	Grade 4 or 5 Non-AER Reportable Events/Hospitalization Form												X	

Add 2,8
1. If a patient is alive 2 years after registration, no further follow-up is required. **(As of Addendum 8, patients no longer need to be followed)**
2. Weekly.
3 The research blood sample and the UGT1A1 genotyping sample is the same sample however the patient must consent to the research portion.

Add 2
4. Submit the blood sample by overnight express to MCLCT (see Section 14.33).

Add 4,5
5. This research blood is collected only on Cycle 1, day 1. See Section 14.3 for sample requirements.

19.0 Budget Considerations:

19.1 Costs charged to patient: Routine clinical care.

19.2 Tests to be research funded: Investigational stains, CK7 and CK20 profile, will be paid for by the NCCTG Chairmen's Fund. These tests will be research funded at a cost of approximately $5 per stain.

Add 3
19.3 Eli Lilly and Company will provide gemcitabine (Gemzar®) for this study.

19.4 Other budget concerns: None.

20.0 References

1. Greco, FA, Hainsworth, JD. Cancer of unknown primary site, in De Vita: Cancer,Principles and Practice of Oncology (5th ed, 1997) pp 2423-2445.

2. Hainsworth, JD & Greco, FA. Management of Patient with Cancer of Unknown Primary Site. Oncology 14 (4): 563-74, 2000.

3. Hainsworth, JD. et al. Carcinoma of unknown primary site: Treatment with 1 hour paclitaxel, carboplatin, and extended-schedule etoposide. JCO 15: 2385-2393, 1997.

4. Pavlidis, N. et al. Cisplatin/Taxol combination chemotherapy in 72 patients with metastatic cancer of unknown primary site: A phase II trial of the Hellenic Cooperative Oncology Group. Proc ASCO 18: 195a, 1999.

5. Noble, S. et al. Gemcitabine. A review of its pharmacology and clinical potential in NSCLC and pancreatic cancer. Drugs. 54: 447-72, 1997.

6. Abratt, RP. Efficacy and safety profile of gemcitabine in NSCLC: A Phase II study. JCO 12: 1535-40, 1994.

7. Fossella, FV. Maximum-tolerated dose defined for single agent gemcitabine: A phase I dose escalation study in chemotherapy-naïve patients with advanced NSCLC. JCO: 15: 310-6, 1997.

8. Gatzemeier,U. et al. Activity of gemcitabine in patients with NSCLC: A multicenter extended phase II trial. Eur Jo CA 32: 243-28, 1996.

9. Anderson, H. et al. Single agent activity of weekly gemcitabine in advanced NSCLC. A phase II study. JCO 12: 1821-26, 1994.

10. Fukuoka, M et al. Phase II studies of gemcitabine for NSCLC in Japan. Sem Onc 24: S7-46, suppl7, 1997.

11. Burris HA 3rd. Moore MJ. Andersen J, et al. Improvements in survival and clinial benefit with gemcitabine as first-line therapy for patients with advanced pancreas cancer: a randomized trial. JCO 15: 2403-13, 1997.

12. Barton, J. et al. Gemcitabine in the second-line therapy of patients with carcinoma of unknown primary site: Phase II trial of the Minnie Pearl Cancer Research Network. Proc ASCO 18: 450a, 1999.

13. Greco FA, Burris HA, Litchy S. et al. Gemcitabine, carboplatin and paclitaxel for patients with carcinoma of unknown primary: A Minnie Pearl Cancer Research network study. JCO 20:1651-56, 2002.

14. Kunimoto, T. et al. Anti-tumor activity of irinotecan,a novel water soluble derivative of camptothecan, against murine tumors. Cancer Res 47: 5944-47, 1987.

15. Kawato, Y. et al. Intracellular roles of SN-38, a metabolite of the camptothecan derivative, irinotecan, in the anti-tumor effect of irinotecan. Cancer Res 51: 4187-91, 1991.

16. Baker et al. Phase II Study of irinotecan in advanced NSCLC. Proc ASCO 16: 461, 1658, 1997.

17. Fukuoka,M. et al. Phase II study of irinotecan, a new derivative of camptothecan, for previously untreated NSCLC. JCO 10: 16-20, 1992.

18. Negoro,S. et al. Phase I study of weekly intravenous infusions of irinotecan JNCI 83: 1164-68, 1991.

19. Douillard et al. Phase II study of irinotecan in NSCLC. Proc ASCO 14: 365 1118, 1995.

20. Rosen, Lee. Irinotecan in Lymphoma, Leukemia, and Breast, Pancreatic, Ovarian, and Small-Cell Lung Cancers. Oncology. 12 (8) Suppl 6: 103-9, 1998.

21. Kanzawa, F. In vitro interaction between gemcitabine and other anticancer drugs using a novel three-dimensional model. Sem Onc 24: S7-16 (Suppl 7), 1997.

22. Bahadori, HR et al. Evaluation of irinotecan and gemcitabine as single agents and in combination in small cell lung cancer cells. Proc ASCO 17: 477 #1837, 1998.

23. Rocha Lima, C. et al. Single-agent gemcitabine and gemcitabine/irinotecan combination in NSCLC. Sem Onc 26(5): 43-50 Suppl 16, 1999.

24. O'Reilly, E. et al. A Phase I study of Combination gemcitabine and irinotecan in patients with refractory solid tumors. Proc ASCO 18: 176a, #674, 1999.

~~25. Alberts, S. et al. Phase I trial of gemcitabine and CPT 11 given weekly for 4 weeks every 6 weeks. Not yet published, 2000.~~

26. Rocha Lima, C. Multicenter Phase II trial of First-line inrinotecan and gemcitabine in patients with locally advanced or metastatic pancreatic cancer. Proc ASCO. 263a #1023, 2000.

27. Stathopoulos, G et al. Front-line treatment of pancreatic carcinoma with gemcitabine in combination with irinotecan: Preliminary results of a multicenter Phase II study. Proc ASCO 319a #1260, 2000.

28. Chu PG, Weiss LM. Keratin expression in human tissues and neoplasms. Histopathology 40:403-439, 2002.

29. Kaneda, N., H. Nagata, et al. (1990). "Metabolism and pharmacokinetics of the camptothecin analogue CPT-11 in the mouse." Cancer Res **50**(6): 1715-20.

30. Kawato, Y., M. Aonuma, et al. (1991). "Intracellular roles of SN-38, a metabolite of the camptothecin derivative CPT-11, in the antitumor effect of CPT-11." Cancer Res **51**(16): 4187-91.

31. Rivory, L. P., J. F. Riou, et al. (1996). "Identification and properties of a major plasma metabolite of irinotecan (CPT-11) isolated from the plasma of patients." Cancer Res **56**(16): 3689-94.

32. Pommier, Y., A. Tanizawa, et al. (1994). "Mechanisms of topoisomerase I inhibition by anticancer drugs." Adv Pharmacol: 73-92.

33. Hsiang, Y. H., M. G. Lihou, et al. (1989). "Arrest of replication forks by drug-stabilized topoisomerase I-DNA cleavable complexes as a mechanism of cell killing by camptothecin." Cancer Res **49**(18): 5077-82.

34. Holm, C., J. M. Covey, et al. (1989). "Differential requirement of DNA replication for the cytotoxicity of DNA topoisomerase I and II inhibitors in Chinese hamster DC3F cells." Cancer Res **49**(22): 6365-8.

35. Shin, C. G. and R. M. Snapka (1990). "Exposure to camptothecin breaks leading and lagging strand simian virus 40 DNA replication forks." Biochem Biophys Res Commun **168**(1): 135-40.

36. Kaufmann, S. H. (1998). "Cell death induced by topoisomerase-targeted drugs: more questions than answers." Biochim Biophys Acta **1400**(1-3): 195-211.

37. Iyer, L., C. D. King, et al. (1998). "Genetic predisposition to the metabolism of irinotecan (CPT-11). Role of uridine diphosphate glucuronosyltransferase isoform 1A1 in the glucuronidation of its active metabolite (SN-38) in human liver microsomes." J Clin Invest **101**(4): 847-54.

38. Iyer, L., D. Hall, et al. (1999). "Phenotype-genotype correlation of in vitro SN-38 (active metabolite of irinotecan) and bilirubin glucuronidation in human liver tissue with UGT1A1 promoter polymorphism." Clin Pharmacol Ther **65**(5): 576-82.

39. Ando, Y., H. Saka, et al. (2000). "Polymorphisms of UDP-glucuronosyltransferase gene and irinotecan toxicity: a pharmacogenetic analysis." Cancer Res **60**(24): 6921-6.

40. Iyer, L., S. Das, et al. (2002). "UGT1A1*28 polymorphism as a determinant of irinotecan disposition and toxicity." Pharmacogenomics J **2**(1): 43-7.

41. McLeod, H. L., D. J. Sargent, et al. (2003). "Pharmacogenetic analysis of systemic toxicity and response after 5-Fluorouracil (5-FU/CPT-11, 5FU/oxaliplatin (oxal) or CPT-11/oxal therapy for advanced colorectal cancer (CRC): Results from an intergroup trial." Proc Am Soc Clin Oncol **22**: 253.

42. Alberts, S. R., C. Erlichman, et al. (2001). "Phase I trial of gemcitabine and CPT-11 given weekly for four weeks every six weeks." Annals of Oncology **12**(5): 627-31.

43. Sugatani, J., K. Yamakawa, et al. (2002). "Identification of a defect in the UGT1A1 gene promoter and its association with hyperbilirubinemia." Biochem Biophys Res Commun **292**(2): 492-7.

44. Innocenti, F., C. Grimsley, et al. (2002). "Haplotype structure of the UDP-glucuronosyltransferase 1A1 promoter in different ethnic groups." Pharmacogenetics **12**(9): 725-733.

45. Innocenti, F. and M. J. Ratain (2002). "Update on pharmacogenetics in cancer chemotherapy." Eur J Cancer **38**(5): 639-44.

46. Illmer, T., U. S. Schuler, et al. (2002). "MDR1 gene polymorphisms affect therapy outcome in acute myeloid leukemia patients." Cancer Res **62**(17): 4955-62.

47. Iyer L, Ramirez J, Shepard DR, Bingham CM, Hossfeld DK, Ratian MJ, et al. (2002) Biliary transport of irinotecan and metabolites in normal and P-glycoprotein-deficient mice. Cancer Chemother Pharmacol; 49(4): 336-41.

48. Mathijssen, R. H., R. J. van Alphen, et al. (2001). "Clinical pharmacokinetics and metabolism of irinotecan (CPT-11)." Clin Cancer Res **7**(8): 2182-94.

49. Akaba, K., T. Kimura, et al. (1998). "Neonatal hyperbilirubinemia and mutation of the bilirubin uridine diphosphate-glucuronosyltransferase gene: a common missense mutation among Japanese, Koreans and Chinese." Biochem Mol Biol Int **46**(1): 21-6.

50. Kaplan E, Meier P: Nonparametric estimation for incomplete observations. J Am Stat Assoc 53:457-481, 1958.

51. Sargent DJ, Chan V, Goldberg RM. A three-outcome design for phase II trials. In press, Controlled Clinical Trials, Oct 2000.

52. Cox DR: Regression models and life tables. J R Stat Soc (Series B) 34:187-202, 1972.

Add 5

53. Therasse P., Arbuck SG, Eisenhauer EA, Wanders J, Kaplan RS, Rubinstein L, Verweij J, Van Glabbeke M, van Oosteron AT, Christian MC, Gwyther SG. New guidelines to evaluate the response to treatment in solid tumors. J Natl Cancer Inst 92(3): 205 – 16, 2000)

54. Pavlidis, N., Briasoulis, E., Hainsworth, J., and Greco, F.A. 2003. Diagnostic and therapeutic management of cancer of an unknown primary. *Eur J Cancer* 39:1990-2005.

55. Pusztai, L., Ayers, M., Symmans, A., Damokosh, A., Hess, K., Valero, V., Clark, E., Ross, J.S., Hortobagyi, G.N., and Stec, J. 2003. Emerging science: prospective validation of gene expression profiling-based prediction of complete pathologic response to neoadjuvant paclitaxel/FAC chemotherapy in breast cancer. In *ASCO*. 22.

56. Abbruzzese, J.L., Abbruzzese, M.C., Hess, K.R., Raber, M.N., Lenzi, R., and Frost, P. 1994. Unknown primary carcinoma: natural history and prognostic factors in 657 consecutive patients. *J Clin Oncol* 12:1272-1280.

57. Mackay, B., and Ordonez, N.G. 1993. Pathological evaluation of neoplasms with unknown primary tumor site. *Semin Oncol* 20:206-228.

58. Motzer, R.J., Rodriguez, E., Reuter, V.E., Bosl, G.J., Mazumdar, M., and Chaganti, R.S. 1995. Molecular and cytogenetic studies in the diagnosis of patients with poorly differentiated carcinomas of unknown primary site. *J Clin Oncol* 13:274-282.

59. Abbruzzese, J.L., Abbruzzese, M.C., Lenzi, R., Hess, K.R., and Raber, M.N. 1995. Analysis of a diagnostic strategy for patients with suspected tumors of unknown origin. *J Clin Oncol* 13:2094-2103.

60. Ayoub, J.P., Hess, K.R., Abbruzzese, M.C., Lenzi, R., Raber, M.N., and Abbruzzese, J.L. 1998. Unknown primary tumors metastatic to liver. *J Clin Oncol* 16:2105-2112.

61. Lenzi, R., Hess, K.R., Abbruzzese, M.C., Raber, M.N., Ordonez, N.G., and Abbruzzese, J.L. 1997. Poorly differentiated carcinoma and poorly differentiated adenocarcinoma of unknown origin: favorable subsets of patients with unknown-primary carcinoma? *J Clin Oncol* 15:2056-2066.

62. Varadhachary, G.R., Abbruzzese, J.L., and Lenzi, R. 2004. Diagnostic strategies for unknown primary cancer. *Cancer* 100:1776-1785.

63. Tomaszewski, J.E., and LiVolsi, V.A. 1999. Mandatory second opinion of pathologic slides: is it necessary? *Cancer* 86:2198-2200.

64. Ma et al., 2006. Molecular Classification of Human Cancers Using a 92-Gene Real-Time Quantitative Polymerase Chain Reaction Assay. Arch. Path. Lab. Med., 130:465473.

Add 2 Appendix IA

TITLE: **Cohort I patients:** A Phase II Study of Gemcitabine (GEMZAR) and Irinotecan (CPT-11) in Previously Untreated Patients with Measurable Disease with Unknown Primary Carcinoma (N004E)

PARTICIPANTS:

This is an important form. Please read it carefully. It tells you what you need to know about this research study. If you agree to take part in this study, you need to sign this form. Your signature means that you have been told about the study and what the risks are. Your signature on this form also means that you want to take part in this study.

Why is this research study being done?

This study is being done to:

- Learn the highest safe dose of the chemotherapy drugs gemcitabine (GEMZAR) and irinotecan (CPT-11) that can be given to patients when the main site of the cancer is not known.

- Find out what effects (good and bad) the GEMZAR and CPT-11 have on you and your cancer.

How many people will take part in this research study?

Add 2,4,5,6 The plan is to have approximately 42 people take part in this study.

What will happen in this research study?

Before you start any study treatment, you will have certain tests and procedures to determine that you can take part in this study. The researcher will look at your medical history, you will have a physical exam, your tumor(s) will be evaluated by x-ray or other method, and you'll have standard blood tests. If you are a woman who can become pregnant you will also have a pregnancy test.

Add 2 Furthermore, you will have an additional 10 ml (approximately one tablespoon) blood sample taken prior to starting the study drugs. This sample will be used to get genetic material called deoxyribonucleic acid (DNA) from your blood cells. The DNA will be used to determine the genetic nature of the enzymes (proteins) that play a role in the biologic activity (side effects and anti-cancer activities) and fate (removal) of the study drugs in your body. The goal of these tests is to better understand individual patient differences in
Add 2,6 the biological effects of Gemzar and CPT-11. An initial genetic test for one enzyme involved in the removal of CPT-11 is required and will be done at the beginning of the study.

Add 5,6 You will be given GEMZAR and CPT-11 into a vein on days 1, 8, 15, and 22. GEMZAR will be given over 30 minutes and CPT-11 will be given over 90 minutes starting 30 minutes after the end of GEMZAR. After the four weekly doses, you will then have no treatment for two-weeks. This is called a cycle of treatment. After the two weeks without treatment, you will be tested to see if the treatment is working and to look for side effects from this drug treatment. As long as the treatment is helpful and side effects are not too bad, you may keep getting this treatment.

Add 2 The study doctors would also like to perform a research test on the 1 tablespoon blood sample that is being collected from you. This test is additional genetic testing done to look at the drug levels in your blood and also to understand how your cancer responds to treatment. It is hoped that this will help investigators better

understand your type of cancer. However, you will not receive the results. You can still take part in this treatment study and choose not to have this research test done.

Add 2 This study also has optional laboratory tests that will study small samples of biopsy tissue. The biopsy tissue sample will be from your original biopsy. No additional biopsies will be done to get this tissue. The tissue will be sent to laboratories associated with NCCTG where the tests will be done. These tests will be done in order to understand how your cancer responds to treatment. It is hoped that this will help investigators better understand your type of cancer. The results of these tests will not be sent to you or your doctor and will not be used in planning your care. These tests are for research purposes only and you will not have to pay for them.

Add 2 Please read the following statement and mark your choice:

I permit my blood sample to be used in research blood test described above.

☐ Yes ☐ No Please initial here:____________ Date:_________

Add 2 I permit my tissue sample to be used in research blood test described above.

☐ Yes ☐ No Please initial here:____________ Date:_________

How long will I be in this research study?

You will get treatment with both drugs until they are no longer helpful or if you or your doctor think that the side effects of the treatment are too bad. Your health status will be watched for up to two years after you have stopped getting the drugs.

Are there reasons I might leave this research study early?

Taking part in this research study is your decision. You may decide to stop at any time. You should tell the researcher if you decide to stop and you will be informed if any additional tests may need to be done for your safety.

In addition, the researchers may stop you from taking part in this study at any time if it is in your best interest, if you do not follow the study rules, or if the study is stopped.

Will any biological sample(s) be stored and used in the future by the North Central Cancer Treatment Group (NCCTG)?

Another part of this research study is taking a small sample(s) of your leftover blood and biopsy tissue to be stored for future research studies of cancer. The sample(s) may be stored indefinitely. You have a say in how your stored sample(s) is (are) used in future research. You can still take part in the treatment study without giving your sample(s).

Your sample(s) will be stored safely at NCCTG and will be given a code (rather than your name) when used in research. This code will allow your sample(s) to be used without anyone knowing that it is (they are) your sample(s) just by looking at the label.

Your sample(s) will be used only for research and will not be sold. You will not be paid for allowing your sample(s) to be used in research even though the research done on the sample(s) may help to develop new products in the future.

Sometimes blood and tissue are used for genetic research (research about diseases that are passed on in families). Even if your sample(s) is (are) used for genetic research, the findings will not be linked with your medical records and they will not be given to people outside of the research process.

Please read the following statements and mark your choice:

Add 2 1. I permit my blood sample to be stored and used for future research of cancer:

☐ Yes ☐ No Please initial here:____________ Date:_________

2. I permit my tissue sample to be stored and used for future research of cancer:

☐ Yes ☐ No Please initial here:____________ Date:_________

Add 2 3. I permit my blood sample to be stored and used in future research to learn, prevent, or treat other health problems:

☐ Yes ☐ No Please initial here:____________ Date:_________

4. I permit my tissue sample to be stored and used in future research to learn, prevent, or treat other health problems:

☐ Yes ☐ No Please initial here:____________ Date:_________

If you want your sample(s) destroyed at any time, write to the Secretary of the ___________________ Institutional Review Board ____. NCCTG has the right to end storage of the sample(s) without telling you.

The sample will be the property of NCCTG. Outside researchers may one day ask for a part of your sample for studies now or future studies.

How do outside researchers get the sample?

Researchers from universities, hospitals, and other health organizations do research using tissue. They may call NCCTG and ask for samples for their studies. NCCTG looks at the way that these studies will be done, and decides if any of the samples can be used. NCCTG sends the tissue sample and some information about you to the researcher. NCCTG will not send your name, address, phone number, social security number, or any other identifying information to the researcher. If you allow your sample to be given to outside researchers, it will be given to them with a code number. If researchers outside NCCTG use the sample for future research, they will decide if you will be contacted and, if so, they would have to contact you through the researchers at NCCTG.

Add 2 I permit NCCTG to give my blood sample to outside researchers:

Please mark one box:
☐ Yes ☐ No Please initial here:____________ Date:_________

I permit NCCTG to give my tissue sample to outside researchers:

Add 2 ***Please mark one box:***
☐ Yes ☐ No Please initial here:____________ Date:_________

What are the risks of this research study?

While you are taking part in this study, you are at risk for these side effects. You should talk to the researcher and/or your medical doctor about these side effects. There also may be other side effects that are not known. Side effects may range from mild to life threatening. Other drugs may be given to make side effects less serious and uncomfortable. Many side effects go away shortly after the gemcitabine and irinotecan are stopped, but in some cases side effects can be serious, long lasting, or may never go away. There may be a risk of death.

Gemcitabine (GEMZAR)

Add 6 **Likely risks of gemcitabine** *(events occurring greater than 20% of the time)*
- Decrease in the white blood cells counts (may increase chance of developing an infection).
- Decrease in the platelets (may increase the chance of bruising or bleeding after injury).
- Decrease in the red blood cells (may cause feelings of being tired).
- Nausea (feeling sick to your stomach).
- Vomiting (being sick).
- Flu-like symptoms, including fever, headaches, mild chills, muscle soreness, fatigue, weakness, lethargy, loss of appetite, cough, runny nose, and sweating.
- Changes in liver function tests (tests that show how the liver is working). These changes are usually mild and non-progressive and rarely require stopping treatment with gemcitabine.
- Diarrhea
- Temporary red itchy rash.
- Tingling, prickling, or creeping feeling on the skin.
- Shortness of breath.
- Fluid retention (usually seen as swelling of the hands, feet, or face).

Add 6 **Less likely risks of gemcitabine** *(events occurring less than or equal to 20% of the time)*
- Mild effect on the kidneys (blood or protein in the urine).
- Mild hair loss
- Inflammation of the mucous membranes of the mouth and throat.
- Loss of appetite.
- Sleepiness.
- Agitation (feeling restless).
- Inability to sleep.

Add 6 **Rare but serious risks of gemcitabine** *(events occurring less than 2-3% of the time)*
- Decrease in blood pressure
- Fast or irregular heart beat.
- Severe allergic reactions. Symptoms may include rash, changes in blood pressure, swelling and increased fluid in the tissues, increases in heart rate, difficulty breathing and collapse.
- Severe changes in liver function tests (including changes that may cause jaundice).
- Severe difficulty breathing.
- Severe effects on the kidneys, sometimes leading to kidney failure.
- Heart failure
- Add 2 Confusion.
- Convulsions.
- Coma.
- Blood vessel inflammation and gangrene (death of soft tissue due to lack of blood supply).
- Capillary leak syndrome, which can cause low blood pressure or reduce the amount of oxygen getting to tissue.
- Blistering red sores in the mucous membranes or larger areas of the body.
- Rapid pulse.
- Bronchospasm (similar to wheezing)
- Severe skin reactions, including peeling of the skin.
- Add 6 Anemia
- Rapid pulse
- Add 2 Lung scarring leading to lung failure

<u>Irinotecan (CPT-11)</u>

Add 7 **Likely risks of Irinotecan (CPT-11) (events occurring greater than 20% of the time)**

- Loose stools. In an effort to lessen the severity of loose stools, you will be asked to count the number of your stools and will be given the drug loperamide to take immediately when the loose stools begin or when you have more than normal stools. If the high number of stools or loose stools do not get better, your doctor should be called.
- Fall of the white blood cell count that can lead to higher risk of infection and bleeding.
- Low platelet count (thrombocytopenia) that may lead to bleeding or easy bruising
- Low red blood cell count (anemia) that may make you feel tired and short of breath; a transfusion may be needed
- Feeling sick to your stomach
- Throwing up
- Sores (ulcers) of the bowels
- Trouble passing stools (constipation)
- Loss of appetite
- Sore mouth
- Hair loss
- Trouble breathing or shortness of breath. In some cases, this has caused death.
- Cough
- General discomfort

Add 7 **Less likely risks of Irinotecan (CPT-11) (events occurring less than or equal to 20% of the time)**

- Being very tired
- Weakness
- Change in liver or kidney function
- Skin rash
- Fever
- Heavy sweating
- Moistness (saliva) of mouth
- Watery eyes
- Slower heart beat
- Leaking of the drug from the IV area may cause sores on the skin or very bad redness and soreness.
- Flushing or a warm sensation
- Lower blood pressure, which may cause lightheadedness or dizziness, or may cause you to pass out

Add 7 **Rare but serious risks of Irinotecan (CPT-11 (events occurring less than 2-3% of the time)**

- Loose stools that happen at a time when the white blood cell count is low can be especially bad. There have been deaths reported from these two side effects together. Although the risk of death is low, this shows you how important it is to call the doctor or nurse about any side effects you are having.
- Increased pancreas enzymes levels which can lead to pancreatitis, a condition where the pancreas becomes inflamed and can cause pain

There is not enough medical information to know what the risks might be to a breast-fed infant or to an unborn child of a man or woman who takes part in this study. Men who are able to father a child and women who can become pregnant must use one of these birth control plans during this study: diaphragm, birth control pills, injections, intrauterine device (IUD), surgical sterilization, under the skin implants, abstinence. Another choice is for your sexual partner to use one of these birth control plans. Breast-feeding mothers must stop breast-feeding to take part in this study. Women who can become pregnant must have a pregnancy test before taking part in this study. For the pregnancy test, blood will be taken from a vein in your arm with a needle within 7 days before you enter the study. You will be told if you are pregnant or not. If you are pregnant, you will not be able to take part in the study.

Are there benefits to taking part in this research study?

This study may not make your health better. However, there is a chance that these drugs may make the cancer better. It is not known if this combination of drugs is better than standard types of chemotherapy.

What other choices do I have if I don't take part in this research study?

You do not have to be in this study to receive treatment for your condition. Your other choices may include standard types of chemotherapy or supportive care only. You may also decide to take part in another type of clinical trial if you are eligible. You should talk to your doctor about each of your choices before you decide if you will take part in this study.

Will I need to pay for the tests and procedures?

Add 3 Irinotecan can be bought with a prescription. Eli Lilly will provide you with the gemcitabine free of charge for this study. Every effort has been made to ensure adequate supplies of gemcitabine, free of charge, for all who take part. If, however, gemcitabine is approved by the Food and Drug Administration (FDA) for use in cancers where the primary site is not known, while you are being treated, there is a possibility that you and/or your health plan would be asked to purchase subsequent supplies.

Add 3 You and/or your health plan will need to pay for all tests and procedures that are part of this study because they are needed for your regular medical care. You and/or your health plan may also have to pay for other drugs or treatment that are given to help control side effects as well as the cost of tests or exams to evaluate possible side effects. Before you take part in this study, you should call your health insurer to find out if the cost of these tests and/or procedures will be paid for by the plan. Some health insurers will not pay for these costs. You will have to pay for any costs not covered by your health insurer.

What happens if I am injured because I took part in this research study?

If you have side effects from the study treatment, you need to report them to the researcher and your regular physician, and you will be treated as needed. You and your insurer will be billed for these services at the usual charge. You will not be offered free medical care or payment for any bad side effects from taking part in this study. Medical services will be given at the usual charge.

What are my rights if I take part in this research study?

Taking part in this research study does not take away any other rights or benefits you might have if you did not take part in the study. Taking part in this study does not give you any special privileges. You will not be penalized in any way if you decide not to take part or if you stop after you start the study. Specifically, you do not have to be in this study to receive or continue to receive medical care. If you stop the study you would still receive medical care for your condition although you might not be able to get the study treatment. You will be told of important new findings or any changes in the study or procedures that may affect you or your willingness to continue in the study.

Who can answer my questions?

You may talk to Dr. (_______________), telephone (______________________), at any time about any question you have on this study.

You can get information about policies, the conduct of the study, or the rights of research subjects from (____
____)

Where can I get more information about research studies?

You may call the NCI's Cancer Information Service at 1-800-4-CANCER (1-800-422-6237) or TTY: 1-800-332-8615

Visit the NCI Web site: http://www.cancer.gov/

What about confidentiality?

Information from this study may be published or presented at scientific meetings. However, your name and other identifying information will not be sent outside of NCCTG without written permission unless the law allows it. Your medical record will be used by the researchers in this study. Representatives of NCCTG will be able to look at your medical records to check the accuracy of the forms completed for the study.
Add 2,3 Information from your medical records may also be made available to Eli Lilly and Company, the Food and Drug Administration (FDA), National Cancer Institute (NCI), other U.S. government agencies including the Office for Human Research Protections or other offices within the Department of Health and Human Services, and/or the Office of the Inspector General.

I have had an opportunity to have my questions answered. I have been given a copy of this form. I agree to participate in this study.

__________ ____
(Date) (Printed Name of Participant)

____
(Signed Name of Participant)

__________ ____
(Date) (Printed Name of Individual Obtaining Consent)

____
(Signed Name of Individual Obtaining Consent)

Local IRB changes to this document are allowed. Sections "What Are The Risks Of The Study" or "What Other Choices Do I Have If I Don't Take Part In This study?" should always be tried to be used in their entirety. Editorial changes to these sections may be made as long as they do not change information or intent. If the institutional IRB insists on making deletions or more substantive modifications to these sections, they may be justified in writing by the investigator and approved by the IRB. Under these circumstances, the revised language and justification must be forwarded to the North Central Cancer Treatment Group Operations Office for approval before a patient may be registered to this study.

Consent forms will have to be modified for each institution as it relates to where information may be obtained on the conduct of the study or research subject. This should be specific for each institution.

Add 2

Appendix IB

TITLE: **Cohort II patients:** A Phase II Study of Gemcitabine (GEMZAR) and Irinotecan (CPT-11) in Previously Untreated Patients with Measurable Disease with Unknown Primary Carcinoma (N004E)

PARTICIPANTS:

This is an important form. Please read it carefully. It tells you what you need to know about this research study. If you agree to take part in this study, you need to sign this form. Your signature means that you have been told about the study and what the risks are. Your signature on this form also means that you want to take part in this study.

Why is this research study being done?

This study is being done to:

- Learn the highest safe dose of the chemotherapy drugs gemcitabine (GEMZAR) and irinotecan (CPT-11) that can be given to patients when the main site of the cancer is not known.
- Find out what effects (good and bad) the GEMZAR and CPT-11 have on you and your cancer.

How many people will take part in this research study?

Add 2,4,5,6 The plan is to have a total of approximately 42 people take part in this study. Fourteen patients were enrolled in the first part of this study (Cohort I). About 28 patients will be enrolled during the second part of the study (Cohort II) based upon the results of a genetic test for one enzyme involved in the removal of CPT-11.

What will happen in this research study?

Before you start any study treatment, you will have certain tests and procedures to determine that you can take part in this study. The researcher will look at your medical history, you will have a physical exam, your tumor(s) will be evaluated by x-ray or other method, and you'll have standard blood tests. If you are a woman who can become pregnant you will also have a pregnancy test.

Add 2,4 Furthermore, you will have an additional 10 ml (approximately one tablespoon) blood sample taken prior to starting the study drugs. This sample will be used to get genetic material called deoxyribonucleic acid (DNA) from your blood cells. The DNA will be used to determine the genetic nature of the enzymes (proteins) that play a role in the biologic activity (side effects and anti-cancer activities) and fate (removal) of the study drugs (both gemcitabine and CPT-11) in your body. The goal of these tests is to better understand individual patient differences in the biological effects of Gemzar and CPT-11. This test is required and will
Add 2,4,6 be done at the beginning of the study.

You will be given GEMZAR and CPT-11 into a vein on days 1, 8, and 15. GEMZAR will be given over 30
Add 6 minutes and CPT-11 will be given over 90 minutes starting 30 minutes after the end of GEMZAR. More research blood samples will be taken at the following times: 1) at the end of the GEMZAR infusion; and 2) 60 minutes after the GEMZAR infusion is completed. After the three weekly doses, you will then have no treatment for 1 week. This is called a cycle of treatment. After the 1 week without treatment, you will be tested to see if the treatment is working and to look for side effects from this drug treatment. As long as the treatment is helpful and side effects are not too bad, you may keep getting this treatment.

Add 2,4 The study will also perform a research test using a 1-tablespoon blood sample that will be collected prior to the gemcitabine infusion, at the end of infusion and 60 minutes after the infusion. This test is being performed so that researchers better understand how the body processes gemcitabine. You will not receive the results of this study. This test is required for study entry.

Add 5 This study also has laboratory tests that will study small samples of biopsy tissue. The biopsy tissue sample will be from your original biopsy. No additional biopsies will be done to get this tissue.
The tissue will be sent to laboratories associated with NCCTG where the tests will be done. These tests will be done in order to understand how your cancer responds to treatment. It is hoped that this will help investigators better understand your type of cancer. The results of these tests will not be sent to you or your doctor and will not be used in planning your care. These tests are for research purposes only and you will not have to pay for them.

How long will I be in this research study?

You will get treatment with both drugs until they are no longer helpful or if you or your doctor think that the side effects of the treatment are too bad. Your health status will be watched for up to two years after you have stopped getting the drugs.

Are there reasons I might leave this research study early?

Taking part in this research study is your decision. You may decide to stop at any time. You should tell the researcher if you decide to stop and you will be informed if any additional tests may need to be done for your safety.

In addition, the researchers may stop you from taking part in this study at any time if it is in your best interest, if you do not follow the study rules, or if the study is stopped.

Will any biological sample(s) be stored and used in the future by the North Central Cancer Treatment Group (NCCTG)?

Another part of this research study is taking a small sample of your leftover blood and biopsy tissue to be stored for future research studies of cancer. The sample(s) may be stored indefinitely. You have a say in how your stored sample(s) is (are) used in future research. You can still take part in the treatment study without giving your sample(s).

Your sample(s) will be stored safely at NCCTG and will be given a code (rather than your name) when used in research. This code will allow your sample(s) to be used without anyone knowing that it is (they are) your sample(s) just by looking at the label.

Your sample(s) will be used only for research and will not be sold. You will not be paid for allowing your sample(s) to be used in research even though the research done on the sample(s) may help to develop new products in the future.

Sometimes blood and tissue are used for genetic research (research about diseases that are passed on in families). Even if your sample(s) is (are) used for genetic research, the findings will not be linked with your medical records and they will not be given to people outside of the research process.

Please read the following statements and mark your choice:

Add 2 1. I permit my blood sample to be stored and used for future research of cancer*:*

☐ Yes ☐ No Please initial here:___________ Date:_________

2. I permit my tissue sample to be stored and used for future research of cancer:

☐ Yes ☐ No Please initial here:___________ Date:_________

Add 2 3. I permit my blood sample to be stored and used in future research to learn, prevent, or treat other health problems:

☐ Yes ☐ No Please initial here:___________ Date:_________

4. I permit my tissue sample to be stored and used in future research to learn, prevent, or treat other health problems:

☐ Yes ☐ No Please initial here:___________ Date:_________

If you want your sample(s) destroyed at any time, write to the Secretary of the __________________ Institutional Review Board ____. NCCTG has the right to end storage of the sample(s) without telling you.

The sample will be the property of NCCTG. Outside researchers may one day ask for a part of your sample for studies now or future studies.

How do outside researchers get the sample?

Researchers from universities, hospitals, and other health organizations do research using tissue. They may call NCCTG and ask for samples for their studies. NCCTG looks at the way that these studies will be done, and decides if any of the samples can be used. NCCTG sends the tissue sample and some information about you to the researcher. NCCTG will not send your name, address, phone number, social security number, or any other identifying information to the researcher. If you allow your sample to be given to outside researchers, it will be given to them with a code number. If researchers outside NCCTG use the sample for future research, they will decide if you will be contacted and, if so, they would have to contact you through the researchers at NCCTG.

Add 2 I permit NCCTG to give my blood sample to outside researchers:

Please mark one box:
☐ Yes ☐ No Please initial here:___________ Date:_________

Add 2 I permit NCCTG to give my tissue sample to outside researchers:

Please mark one box:
☐ Yes ☐ No Please initial here:___________ Date:_________

What are the risks of this research study?

While you are taking part in this study, you are at risk for these side effects. You should talk to the researcher and/or your medical doctor about these side effects. There also may be other side effects that are not known. Side effects may range from mild to life threatening. Other drugs may be given to make side effects less serious and uncomfortable. Many side effects go away shortly after the gemcitabine and irinotecan are stopped, but in some cases side effects can be serious, long lasting, or may never go away. There may be a risk of death.

<u>**Gemcitabine (GEMZAR)**</u>

Add 6 **Likely risks of gemcitabine** *(events occurring greater than 20% of the time)*
- Decrease in the white blood cells counts (may increase chance of developing an infection).
- Decrease in the platelets (may increase the chance of bruising or bleeding after injury).
- Decrease in the red blood cells (may cause feelings of being tired).
- Nausea (feeling sick to your stomach).
- Vomiting (being sick).
- Flu-like symptoms, including fever, headaches, mild chills, muscle soreness, fatigue, weakness, lethargy, loss of appetite, cough, runny nose, and sweating.
- Changes in liver function tests (tests that show how the liver is working). These changes are usually mild and non-progressive and rarely require stopping treatment with gemcitabine.
- Diarrhea
- Temporary red itchy rash.
- Tingling, prickling, or creeping feeling on the skin.
- Shortness of breath.
- Fluid retention (usually seen as swelling of the hands, feet, or face).

Add 6 **Less likely risks of gemcitabine** *(events occurring less than or equal to 20% of the time)*
- Mild effect on the kidneys (blood or protein in the urine).
- Mild hair loss
- Inflammation of the mucous membranes of the mouth and throat.
- Loss of appetite.
- Sleepiness.
- Agitation (feeling restless).
- Inability to sleep.

Add 6 **Rare but serious risks of gemcitabine** *(events occurring less than 2-3% of the time)*
- Decrease in blood pressure
- Fast or irregular heart beat.
- Severe allergic reactions. Symptoms may include rash, changes in blood pressure, swelling and increased fluid in the tissues, increases in heart rate, difficulty breathing and collapse.
- Severe changes in liver function tests (including changes that may cause jaundice).
- Severe difficulty breathing.
- Severe effects on the kidneys, sometimes leading to kidney failure.
- Heart failure
- Add 2 Confusion.
- Convulsions.
- Coma.
- Blood vessel inflammation and gangrene (death of soft tissue due to lack of blood supply).
- Capillary leak syndrome, which can cause low blood pressure or reduce the amount of oxygen getting to tissue.
- Blistering red sores in the mucous membranes or larger areas of the body.
- Bronchospasm (similar to wheezing)
- Severe skin reactions, including peeling of the skin.
- Add 6 Anemia
- Rapid pulse
- Add 2 Lung scarring leading to lung failure

Irinotecan (CPT-11)

Add 7 **Likely risks of Irinotecan (CPT-11) (events occurring greater than 20% of the time)**

- Loose stools. In an effort to lessen the severity of loose stools, you will be asked to count the number of your stools and will be given the drug loperamide to take immediately when the loose stools begin or when you have more than normal stools. If the high number of stools or loose stools do not get better, your doctor should be called.
- Fall of the white blood cell count that can lead to higher risk of infection and bleeding.
- Low platelet count (thrombocytopenia) that may lead to bleeding or easy bruising
- Low red blood cell count (anemia) that may make you feel tired and short of breath; a transfusion may be needed
- Feeling sick to your stomach
- Throwing up
- Sores (ulcers) of the bowels
- Trouble passing stools (constipation)
- Loss of appetite
- Sore mouth
- Hair loss
- Trouble breathing or shortness of breath. In some cases, this has caused death.
- Cough
- General discomfort

Add 7 **Less likely risks of Irinotecan (CPT-11) (events occurring less than or equal to 20% of the time)**

- Being very tired
- Weakness
- Change in liver or kidney function
- Skin rash
- Fever
- Heavy sweating
- Moistness (saliva) of mouth
- Watery eyes
- Slower heart beat
- Leaking of the drug from the IV area may cause sores on the skin or very bad redness and soreness.
- Flushing or a warm sensation
- Lower blood pressure, which may cause lightheadedness or dizziness, or may cause you to pass out

Add 7 **Rare but serious risks of Irinotecan (CPT-11 (events occurring less than 2-3% of the time)**

- Loose stools that happen at a time when the white blood cell count is low can be especially bad. There have been deaths reported from these two side effects together. Although the risk of death is low, this shows you how important it is to call the doctor or nurse about any side effects you are having.
- Increased pancreas enzymes levels which can lead to pancreatitis, a condition where the pancreas becomes inflamed and can cause pain

There is not enough medical information to know what the risks might be to a breast-fed infant or to an unborn child of a man or woman who takes part in this study. Men who are able to father a child and women who can become pregnant must use one of these birth control plans during this study: diaphragm, birth control pills, injections, intrauterine device (IUD), surgical sterilization, under the skin implants, abstinence. Another choice is for your sexual partner to use one of these birth control plans. Breast-feeding mothers must stop breast-feeding to take part in this study. Women who can become pregnant must have a pregnancy test before taking part in this study. For the pregnancy test, blood will be taken from a vein in your arm with a needle within 7 days before you enter the study. You will be told if you are pregnant or not. If you are pregnant, you will not be able to take part in the study.

Are there benefits to taking part in this research study?

This study may not make your health better. However, there is a chance that these drugs may make the cancer better. It is not known if this combination of drugs is better than standard types of chemotherapy.

What other choices do I have if I don't take part in this research study?

You do not have to be in this study to receive treatment for your condition. Your other choices may include standard types of chemotherapy or supportive care only. You may also decide to take part in another type of clinical trial if you are eligible. You should talk to your doctor about each of your choices before you decide if you will take part in this study.

Will I need to pay for the tests and procedures?

Irinotecan can be bought with a prescription. Eli Lilly will provide you with the gemcitabine free of charge for this study. Every effort has been made to ensure adequate supplies of gemcitabine, free of charge, for all
Add 3 who take part. If, however, gemcitabine is approved by the Food and Drug Administration (FDA) for use in cancers where the primary site is not known, while you are being treated, there is a possibility that you and/or your health plan would be asked to purchase subsequent supplies.

You and/or your health plan will need to pay for all tests and procedures that are part of this study because they are needed for your regular medical care. You and/or your health plan may also have to pay for other
Add 3 drugs or treatment that are given to help control side effects as well as the cost of tests or exams to evaluate possible side effects. Before you take part in this study, you should call your health insurer to find out if the cost of these tests and/or procedures will be paid for by the plan. Some health insurers will not pay for these costs. You will have to pay for any costs not covered by your health insurer.

What happens if I am injured because I took part in this research study?

If you have side effects from the study treatment, you need to report them to the researcher and your regular physician, and you will be treated as needed. You and your insurer will be billed for these services at the usual charge. You will not be offered free medical care or payment for any bad side effects from taking part in this study. Medical services will be given at the usual charge.

What are my rights if I take part in this research study?

Taking part in this research study does not take away any other rights or benefits you might have if you did not take part in the study. Taking part in this study does not give you any special privileges. You will not be penalized in any way if you decide not to take part or if you stop after you start the study. Specifically, you do not have to be in this study to receive or continue to receive medical care. If you stop the study you would still receive medical care for your condition although you might not be able to get the study treatment.

You will be told of important new findings or any changes in the study or procedures that may affect you or your willingness to continue in the study.

Who can answer my questions?

You may talk to Dr. (_______________), telephone (______________________), at any time about any question you have on this study.

You can get information about policies, the conduct of the study, or the rights of research subjects from (____ _________________________________)

Where can I get more information about research studies?

You may call the NCI's Cancer Information Service at 1-800-4-CANCER (1-800-422-6237) or TTY: 1-800-332-8615

Visit the NCI Web site: http://www.cancer.gov/

What about confidentiality?

Information from this study may be published or presented at scientific meetings. However, your name and other identifying information will not be sent outside of NCCTG without written permission unless the law allows it. Your medical record will be used by the researchers in this study. Representatives of NCCTG will be able to look at your medical records to check the accuracy of the forms completed for the study.
Add 2,3 Information from your medical records may also be made available to Eli Lilly and Company, the Food and Drug Administration (FDA), National Cancer Institute (NCI), other U.S. government agencies including the Office for Human Research Protections or other offices within the Department of Health and Human Services, and/or the Office of the Inspector General.

I have had an opportunity to have my questions answered. I have been given a copy of this form. I agree to participate in this study.

__________ ____
(Date) (Printed Name of Participant)

____
(Signed Name of Participant)

__________ ____
(Date) (Printed Name of Individual Obtaining Consent)

____
(Signed Name of Individual Obtaining Consent)

Local IRB changes to this document are allowed. Sections "What Are The Risks Of The Study" or "What Other Choices Do I Have If I Don't Take Part In This study?" should always be tried to be used in their entirety. Editorial changes to these sections may be made as long as they do not change information or intent. If the institutional IRB insists on making deletions or more substantive modifications to these sections, they may be justified in writing by the investigator and approved by the IRB. Under these circumstances, the revised language and justification must be forwarded to the North Central Cancer Treatment Group Operations Office for approval before a patient may be registered to this study.

Consent forms will have to be modified for each institution as it relates to where information may be obtained on the conduct of the study or research subject. This should be specific for each institution.

PLACE LABEL HERE

Protocol # N004E

Patient ID # ____________ Initials: ____________
L F M

Local ID # ____________ Institution ____________

NORTH CENTRAL CANCER TREATMENT GROUP

APPENDIX II

PATIENT INSTRUCTIONS FOR PREVENTING AND TREATING DIARRHEA

Be aware of your bowel movements. At the first signs that stools are softer than usual, runny or watery, or if you have more bowel movements in a day than normal, begin taking loperamide. **If you do not start taking loperamide right away, severe diarrhea may occur. This can last several days and require hospitalization.** Please follow these directions carefully.

- Take two caplets (4 mg) at the first signs of any changes (see above).
- Continue taking one capsule (2 mg) every two hours until you have returned to your normal pattern of bowel movements for 12 hours. Start taking loperamide again (same doses and frequency) if diarrhea returns.
- During the night, you may take two capsules every four hours instead.
- If you have diarrhea, stop taking any laxatives and avoid dairy products.
- Please call your doctor if you have any questions about taking loperamide, if your diarrhea is not under control after three days, if you are feeling extremely weak, or if you think you are having any side effects.
- Be sure to drink plenty of fluids each day (several glasses of water, fruit juice, soda, soup, etc.). This will help prevent dehydration (note: fluids do **not** cause diarrhea).
- Side effects of loperamide may include tiredness, drowsiness or dizziness. If you experience these effects, avoid driving motorized vehicles or operating machinery.
